# Trust in science, knowledge and risk perception as predictors of COVID-19 vaccination: application of an extended Theory of Planned Behavior model in Hungary

**DOI:** 10.1186/s12889-026-26421-5

**Published:** 2026-02-03

**Authors:** Marianna Kopasz, Zsófia Papp, Csilla Zsigmond, Ildikó Husz

**Affiliations:** 1 ELTE Centre for Social Sciences, Tóth Kálmán u. 4, Budapest, H-1097 Hungary; 2 Corvinus Institute for Advanced Studies, Corvinus University of Budapest, Budapest, Hungary; 3 Department of Sociology and Social Policy, University of Debrecen, Debrecen, Hungary

**Keywords:** COVID-19 vaccination, Trust in science, Knowledge about COVID-19, Perceived risk of COVID-19, Theory of planned behavior, Hungary

## Abstract

**Background:**

Although a number of studies have shown that some form of trust plays an important role in vaccination decisions, few studies have explicitly examined trust in science. This study aims to explore the determinants of COVID-19 vaccine acceptance, with special regard to trust in science. For our empirical analysis, we focus on Hungary, a case characterized by high infection and mortality rates during the pandemic, as well as relatively low levels of trust in science. The study’s contribution to the literature is that, first, in contrast to most previous studies, it builds on an established theoretical framework, and second, within this framework, it analyses the impact of trust in science in relation to knowledge and perceived risk of COVID-19. We use the Theory of Planned Behavior (TBP) to better understand predictors of COVID-19 vaccine acceptance. The key components of TBP (i.e. attitudes, subjective norms, and perceived behavioral control) are extended with further predictors of attitudes toward COVID-19 vaccination: trust in science, knowledge about COVID-19, and perceived risk of COVID-19. To our knowledge, ours is the first study using TPB to examine the impact of these three factors on attitudes towards vaccination in a single model.

**Methods:**

To test our extended TPB model, we use a sample of 761 Hungarian adults from a cross-sectional survey conducted in late 2022. We apply structural equation modeling (SEM) with COVID-19 vaccine acceptance as the main endogenous variable.

**Results:**

Our findings show that of the key components of TPB, attitude toward COVID-19 vaccination is the strongest predictor of vaccine acceptance. Perceived COVID-19 risk, COVID-19-related knowledge, and trust in science are all important predictors of attitude toward COVID-19 vaccination. Importantly, perceived risk and knowledge are also related to trust in science in a meaningful way.

**Conclusions:**

Our findings suggest that since COVID-19-related knowledge and trust in science are interrelated, vaccination campaigns cannot focus on only one or the other: knowledge without trust and vice versa are not enough to increase vaccine acceptance. However, for those with low levels of education, trust in science does not influence attitudes toward vaccination, so increasing COVID-19-related knowledge is key to fostering pro-vaccine attitudes.

**Supplementary Information:**

The online version contains supplementary material available at 10.1186/s12889-026-26421-5.

## Introduction

As with COVID-19, where vaccine uptake played an important role in mitigating the pandemic [1], vaccination could be a crucial factor in similar health emergencies in the future. Numerous studies examine the causes of vaccine hesitancy—defined as the delay in acceptance or refusal of safe vaccines despite the availability of vaccination services [2]—in both national and comparative contexts. Given the huge variation in vaccine uptake across countries, it is essential to look at the determinants of vaccine uptake in as many contexts as possible. This allows for a better understanding of vaccine hesitancy and, hence, creates opportunities for a more effective and safer management of pandemics.

Our study investigates the determinants of COVID-19 vaccine acceptance, with a special focus on trust in science. A number of works have shown the importance of some type of trust in vaccination decisions, including trust in government [3], the health system [4], or confidence in the vaccine [5]. Despite its apparent importance in the context of the pandemic, trust in science specifically has only been investigated by a small number of studies [6]. With very few exceptions [7, 8], these studies did not rely on any established theoretical framework, and examined the direct effect of trust on vaccine acceptance. We contribute to this literature by using the Theory of Planned Behavior (TPB) [9] to explore the role of trust in science in COVID-19 vaccination. While the TPB originally predicts the *intention* to perform a behavior (i.e. intention to get vaccinated), it is also applicable to predict actual *behavior* (i.e. vaccine acceptance) [10]. In our modified TPB model, COVID-19 vaccine acceptance is the product of three factors: (1) attitudes towards vaccination, (2) subjective norms (i.e. an individual’s perceptions of what significant others think about vaccination), and (3) perceived behavioral control (i.e. perceived difficulty of performing the behavior). In this study, we extend the TPB model with further predictors of attitudes toward vaccination: trust in science, knowledge about COVID-19, and perceived risk of COVID-19. To our knowledge, ours is the first study using TPB to examine the impact of these three factors on attitudes towards vaccination in a single model. Additionally, given the importance of trust in science in COVID-19 vaccine acceptance, our study also aims to explore its determinants so that we can contribute to better-targeted health communication campaigns that aim at both increasing trust and encouraging vaccine uptake.

We formulate our research question as follows: What are the determinants of COVID-19 vaccine acceptance in Hungary, with special regard to the roles of trust in science, knowledge about COVID-19, and perceived risk of COVID-19, as examined through an extended Theory of Planned Behavior (TPB) model? Based on this extended TPB framework, we propose the followings: attitudes toward COVID-19 vaccination, subjective norms, and perceived behavioral control are expected to positively predict vaccine acceptance (H1–H3), while attitudes are further shaped by trust in science, COVID-19-related knowledge, and perceived risk of COVID-19 (H4, H6, H7). In turn, trust in science is hypothesized to be positively influenced by epistemic trust (H5). Together, these hypotheses enable us to test both the direct and indirect pathways through which individual beliefs and trust shape vaccine acceptance.

For our empirical analysis, we focus on Hungary, a case characterized by high infection and mortality rates during the pandemic, as well as relatively low levels of trust in science [11]. The analysis is based on cross-sectional survey data collected in November 2022. As of autumn 2022, Hungary registered more than 2 million COVID-19 cases and almost 50 thousand COVID-related deaths^1^. This put Hungary fifth in the world regarding the number of COVID-19 deaths per 100,000 capita. Even though vaccines became widely accessible by May 2021, at the time of our data collection, only 62% of the population had received at least one dosage. While this figure is on par with the world average (61%), it is within the lowest quarter of the EU^2^. Hungary also demonstrates low levels of trust in science [12], and conspiracy theories easily find their audience [13]. This provides a fertile ground for vaccine skepticism, hampering pandemic-mitigation efforts.

### Theoretical background and hypotheses

#### Theory of Planned Behavior

To examine the factors that determine the intention to get a COVID-19 vaccine, the present study draws on the TPB [9, 14]. The theory posits that the behavioral intention is the outcome of three components: (1) attitudes toward the behavior, (2) subjective norms, and (3) perceived behavioral control. The *attitude toward the behavior* refers to the extent to which an individual has a favorable or unfavorable evaluation of the behavior in question. The theory assumes that the more positive the individual’s attitude toward the behavior, the stronger their intention to perform the particular behavior. S*ubjective norms* refer to an individual’s perceptions of what significant others (e.g. family, friends, co-workers) think about the performance of the behavior. In other words, subjective norms represent the social pressure perceived by the individual to perform or not to perform the behavior in question. The TPB posits that the more favorable the subjective norms for a particular behavior, the stronger the individual’s intention to perform the behavior. *Perceived behavioral control* relates to the perceived difficulty of performing the behavior. According to the TPB, the greater the behavioral control the individual perceives over the intended behavior, the stronger their intention to perform the behavior.

**Fig. 1:**
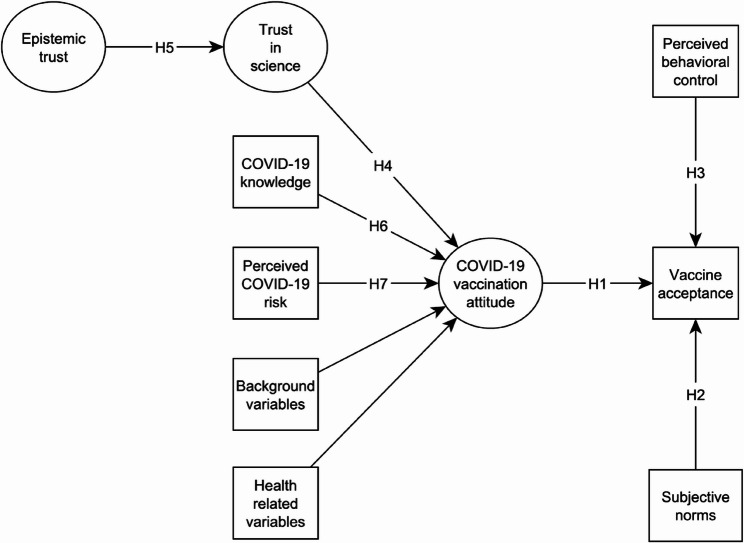
The theoretical model explaining vaccine acceptance

We deviate from the original TPB in that our study focuses on *vaccine acceptance* rather than vaccination intention (see the Methods section). This is justified by the fact that the data for the present study were collected when a large proportion of the population had already been vaccinated. Based on TPB, the following hypotheses were formulated (see Fig. 1 for the proposed theoretical model):

- *H1*: Attitude toward COVID-19 vaccination is positively associated with vaccine acceptance.
- *H2*: Subjective norms regarding COVID-19 vaccination are positively associated with vaccine acceptance.
- *H3*: Perceived behavioral control is positively associated with vaccine acceptance.

In what follows, we extend the TPB model to achieve a more nuanced understanding of vaccine acceptance by including three factors hypothesized to influence attitudes toward vaccination: trust in science, COVID-19–related knowledge, and perceived risk of COVID-19.

#### Trust

Some attempts have been made in the literature to combine TBP with other health behavior theories including the Health Belief Model [8, 15]. Another line of research is to extend TPB with additional constructs – for example, risk perception [1], belief in conspiracy theories [7], or aptitude to misinformation [16] – to better predict the COVID-19 vaccination intention. Our study is related to this latter line of research by complementing the core TPB model with the following components: (1) trust in science, (2) knowledge about COVID-19, and (3) perceived risk of COVID-19. Consistent with TPB, we assume that these further components affect vaccine acceptance *indirectly through attitudes toward COVID-19 vaccination* (i.e. are likely predictors of attitudes toward COVID-19 vaccination).

In this study, *trust in science* is one of the key explanatory variables. Trust is – according to an often used definition – an expectation that the other person or institution “will behave in a way that is beneficial, or at least not harmful, and allows for risks to be taken based on this expectation” [17]. Previous research, not building on theories developed in health psychology, has shown that more trust in scientists and medical experts is positively correlated with COVID-19 vaccine acceptance [18], and negatively correlated with vaccine hesitancy [19, 20]. Two recent single-country studies using the TPB – either alone or in combination with another theory – also indicate that trust in science is crucial in predicting COVID-19 vaccination intention [7, 8]. A possible explanation for this link is that scientists are an important source of information on the risks and prevention of COVID-19, so those who trust them are likely to perceive COVID-19 as a real risk and more likely to accept the preventive measures recommended by them [21]. 

Given the significance of trust in science regarding the COVID-19 vaccination, we also aim to investigate the factors influencing it. We employ the concept of epistemic trust, defined as the individual’s “willingness to accept new information from another as trustworthy, generalizable, and relevant” [22]. Epistemic trust enables individuals to effectively acquire knowledge and integrate new information, supporting their social functioning [23]. Thus, we expect that epistemic trust will be related to more acceptance of science as a source of reliable and relevant information, that is, to more trust in science. The following hypotheses were formulated regarding trust:

- *H4*: Trust in science is positively associated with attitude toward COVID-19 vaccination.
- *H5*: Epistemic trust is positively associated with trust in science.

#### COVID-19-related knowledge and perceived risk of COVID-19

We look at two additional factors that may explain vaccine acceptance based on prior evidence: knowledge about COVID-19 and perceived risk of COVID-19. Some previous findings show that more *knowledge about COVID-19* vaccines is associated with a greater likelihood of COVID-19 vaccine acceptance [24, 25], and a lack of knowledge about vaccines is an important driver of vaccine hesitancy [26]. In addition, a study using an extended TPB model to explain COVID-19 vaccination intention indicates that knowledge concerning COVID-19 vaccines has a positive effect on attitude toward COVID-19 vaccination [1]. It is very likely that those who know more about COVID-19 and the vaccination perceive COVID-19 as a real threat, and accept vaccination as a risk-reducing option.

As for the *perceived risk of COVID-19*, models of health behavior—such as the Health Belief Model [27]—identify perceived risk or severity of a disease as key determinants of preventive health behaviors, including vaccination intention. Dryhurst et al. found that risk perception has a significant effect on protective health behaviors [28]. Similarly, other studies have reported that COVID-19 vaccination intention is positively associated with higher perceived likelihood of infection and greater perceived severity of the disease [29, 30]. Furthermore, recent research indicates a positive relationship between the perception of COVID-19 risk and vaccination intention, mediated by fear of COVID-19 [8]. 

Based on previous findings, we hypothesize the following:

- *H6*: COVID-19 related knowledge is positively associated with attitude toward COVID-19 vaccination.
- *H7*: Perceived risk of COVID-19 is positively associated with attitude toward COVID-19 vaccination.

Finally, our proposed theoretical model (see Fig. 1) also includes the usual socioeconomic and health-related factors: age, gender, education, income, subjective general health, and past flu vaccination.

## Methods

### Data collection

We conducted the empirical analysis using cross-sectional survey data collected in November 2022 from respondents aged 18 and older. The 1,500 respondents were randomly selected using quota sampling of the Hungarian adult population from a carefully maintained online panel.^3^ Although the study sample decreased to 761 individuals due to the list-wise deletion of missing records,^4^ the sample remains representative of age (Mean = 48.8, SD = 15.9), gender (male: 48.8% [*n* = 372], female: 51.2% [*n* = 389]), place of residence (Budapest: 17.5% [*n* = 133], town: 52.1% [*n* = 398], village: 30.3% [*n* = 230]), and education (low: 16% [*n* = 121], middle: 63% [*n* = 474], high: 20% [*n* = 166]).^5^

### Measures

The survey items used in this study are included in Additional file 1. The dependent variable of our model is *COVID-19 vaccine acceptance*. We asked respondents if they had already received a vaccine against COVID-19. We coded responses into a binary variable: responses 1 (‘Yes and I plan to get more shots’), 2 (‘Yes and I don’t plan to get more shots’), and 3 (‘Not yet but I am planning to’) were collapsed into one category (1), and response nr. 4 (‘No and I don’t plan to’)^6^ is recoded to another (0). About 75% (*n* = 570) of the respondents fall into Category 1. This implies that vaccinated individuals are overrepresented in our sample.

Exogenous variables in the main TPB-model are COVID-19 vaccine attitudes, perceived behavioral control, and subjective norms. *COVID-19 vaccine attitude* is a latent factor defined by three indicators, as reported in Table 1 and illustrated in Fig. 1. Higher values indicate more positive attitudes toward vaccination against COVID-19. *Perceived behavioral control* is a manifest variable with higher values suggesting that the individual feels that they could freely decide to get vaccinated (Mean = 4.2, SD = 1.3). We created the *subjective norms* variable (2–10) from two items measuring the approval of family and friends not to get vaccinated by simple addition (Mean = 5.1, SD = 2.9). Higher values indicate greater perceived pressure from the respondent’s social environment.

**Table 1:** Latent factors in the analysis

Latent factor	Survey items	Scale	Cronbach’s alpha	McDonald’s omega	Average variance extracted
COVID-19 vaccine attitudes	ATT1 – COVID-19 vaccines are effective in preventing infection and serious illness. ATT2 – COVID-19 vaccines are safe and their known side effects are negligible. ATT3 – By getting vaccinated, we take responsibility for others.	1: I strongly disagree 7: I strongly agree	0.91	0.91	0.77
Trust in science	T1 – How much do you trust scientists in general when it comes to epidemiological information? T2 – How much do you trust health professionals in general when it comes to epidemiological information? (medical doctors, epidemiological experts) T3 – How much do you trust your GP, doctor or pharmacist regarding epidemiological information?	1: No trust at all 5: Complete trust	0.88	0.89	0.73
Epistemic trust	ET1 – I usually ask people for advice when I have a personal problem. ET2 – I find information easier to trust and absorb when it comes from someone who knows me well. ET3 – If I don’t know what to do, my first instinct is to ask someone whose opinion I value.	1: I strongly disagree 7: I strongly agree	0.8	0.8	0.57

One of the key variables in the analysis is *trust in science*, which is a latent factor of three indicators measuring trust in scientists, health care professionals in general, and the respondent’s own doctors regarding epidemiological information. To measure *epistemic trust*, we constructed a latent variable from three items from the epistemic trust panel of the ‘Epistemic Trust, Mistrust and Credulity Questionnaire’ [31] (see Table 1). In both cases, individuals with more trust score higher.

*COVID-19-related knowledge* is an additive index (0–4) of four items that take 1 if the respondent answered the question correctly, and 0 for an incorrect answer (Mean = 2.8, SD = 1.1). We created the variable *perceived risk of COVID-19* (2–10) by adding together two survey items measuring respondent concern regarding COVID-19 with higher values indicating more concern (Mean = 6.1, SD = 2.2). Measuring past vaccination behavior, we include *flu vaccine uptake* (i.e. the frequency of taking the flu vaccine; 1–4) into our model explaining COVID-19 vaccine attitudes (Mean = 1.8, SD = 1.1). *Subjective general health* is a 5-scale item with higher values indicating better health (Mean = 3.6, SD = 0.9). Finally, background variables include *age*, *gender* (male/female), *education* (low/middle/high) as well as *subjective income* (1–5). A full list of variable descriptions and statistical information is available in Additional file 2.

### Data analysis

To explore our hypotheses, we apply Structural Equation Modeling (SEM). Given that at least one of the endogenous variables is binary, we resort to a robust weighted least squares (WLS) estimator [32]. For the assessment of model fit we observe the Comparative Fit Index – CFI, as well as the Root Mean Square Error of Approximation – RMSEA, and use 0.95 and 0.06 cut-offs respectively [33, 34]. We apply standard 95% confidence intervals and report standardized coefficients. We bootstrap standard errors of the indirect and total effects.

Table 2 depicts correlations within our data based on which we decided to add two additional paths to the structural model. Following from the strong correlations, we estimate paths to trust in science from, first, COVID-19 knowledge, and second, perceived risk of COVID-19 to improve model fit.

**Table 2:** Pearson correlation coefficients between the variables in the analysis

	(1)	(2)	(3)	(4)	(5)	(6)	(7)	(8)	(9)	(10)^a^
(1) COVID-19 vaccine attitudes	1	0.81*	0.20*	0.19*	0.47*	0.62*	0.62*	0.39*	− 0.07	0.36*
(2) Trust in science		1	0.33*	0.21*	0.32*	0.54*	0.52*	0.29*	− 0.00	0.18*
(3) Epistemic trust			1	0.09*	− 0.02	0.08*	0.12*	0.04	0.12*	0.00
(4) Perceived behavioral control				1	− 0.01	0.12*	0.22*	0.06	0.04	0.01*
(5) Subjective norms					1	0.38*	0.33*	0.33*	− 0.11*	0.10*
(6) COVID-19 knowledge						1	0.41*	0.28*	− 0.11*	0.22*
(7) Perceived COVID-19 risk							1	0.35*	− 0.22*	0.19*
(8) Flu vaccine uptake								1	− 0.19*	0.09*
(9) Subjective general health									1	0.01*
(10) COVID-19 vaccine acceptance										1

* *p* < .05

^a^ Eta-squared values

We note that although we are aware that cross-sectional data is not ideal to uncover causal effects, and that counter-arguments may be made with regard to the direction of causality in our model, we use the word ‘effect’ when reporting the results of our model, in line with the language of structural equation modeling [35, 36]. Nevertheless, in other parts of the paper, we tone down the causal language whenever causality may seem speculative.

## Results

### The measurement model

To assess how well the three constructs in Table 1 fit our data we performed Confirmatory Factor Analysis (see Additional file 3). Cronbach’s alpha and McDonald’s omega values indicate great construct reliability. As to convergent validity, all standardized factor loadings are larger than 0.7 [37], and the average variance extracted (AVE) is greater than 0.5 [38], suggesting good internal consistency. The correlation between the constructs remains under 0.85 (r_attitudes_science_ = 0.76, r_attitues_epistemic_ = 0.17, r_science_epistemic_ = 0.29) and indicates good discriminant validity. As to the overall fit of the measurement model, the CFI, and the RMSEA are both well within the acceptable range (0.99 and 0.04 respectively).^7^

### The structural model

**Fig. 2:**
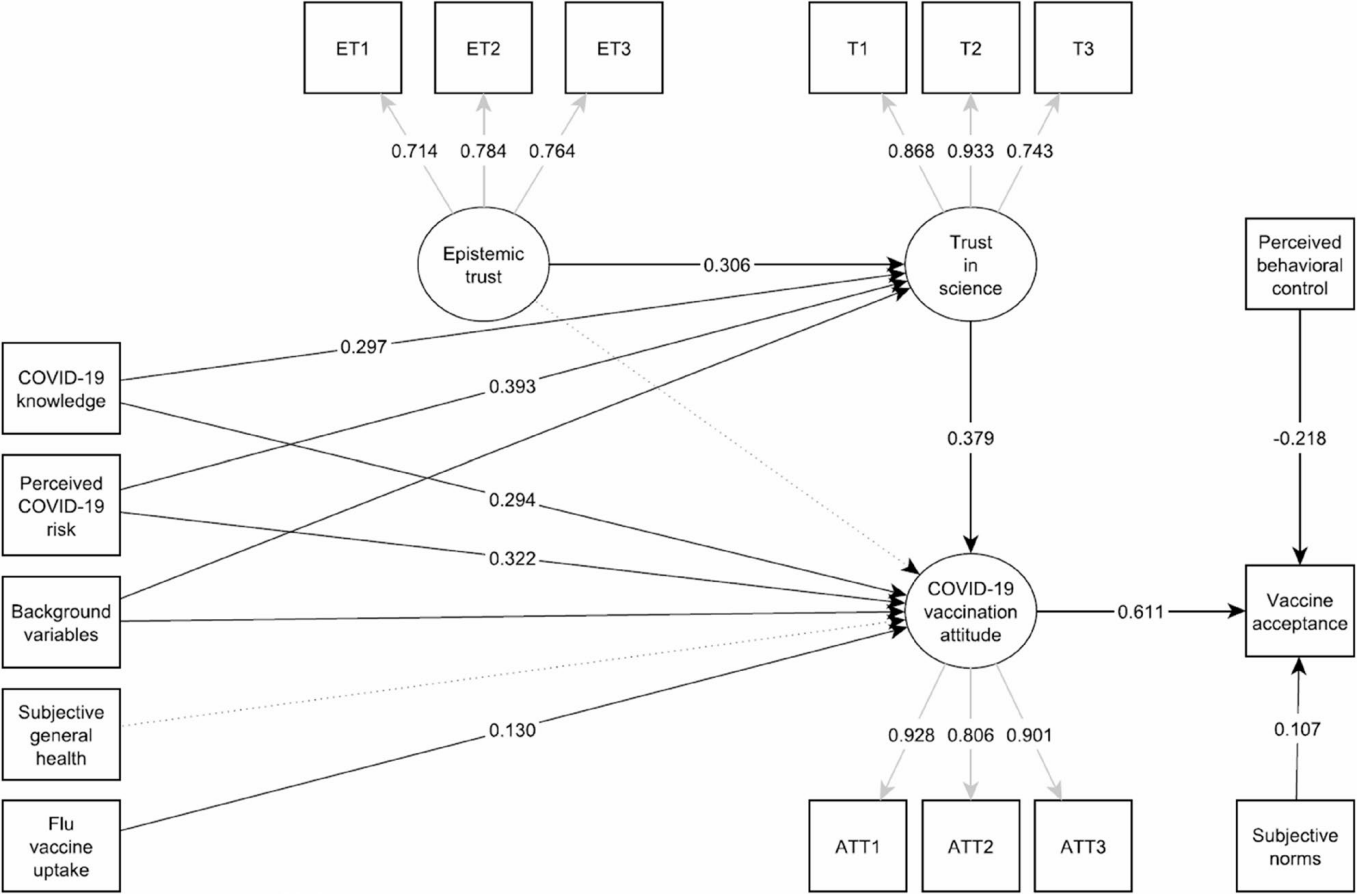
Direct effects in the Structural Equation Model (standardized coefficients)

Figure 2 displays our model with solid lines representing significant effects. Round objects represent latent factors and square objects are manifest variables. For the sake of manageability, we do not visualize estimated variances and covariances (but see Additional file 4). The robust CFI (0.99) and the robust RMSEA (0.04) suggest an adequate fit. Post-hoc investigation puts the power of the analysis above 0.99. We included three endogenous variables in the model: (1) *COVID-19 vaccine acceptance* and (2) *COVID-19 vaccine attitudes*, both within the framework of TPB, and (3) *trust in science* to understand the drivers of our main construct.

### COVID-19 vaccine acceptance

Consistent with the theory, we observe that COVID-19 vaccination attitudes have a positive effect on vaccine acceptance. The effect of subjective norms is also in line with TPB. At the same time, the effect of perceived behavioural control runs in the opposite direction to what the TPB predicts: the stronger an individual’s perceived control over their vaccination behavior, the lower their likelihood of accepting the vaccine.

While within the framework of TPB, only these three variables affect vaccine acceptance directly, as per our SEM various other variables have indirect effects. The effects are mediated through COVID-19 vaccination attitudes and trust in science as indicated by Fig. 2. Three variables appear to affect COVID-19 vaccine uptake to a meaningful extent: COVID-19 knowledge, trust in science, and perceived risk of COVID-19. Epistemic trust and flu vaccine uptake have significant but marginal total effects.

### COVID-19 vaccination attitudes

Moving on to the determinants of COVID-19 vaccination attitudes, we entered background variables (age, gender, level of education and subjective income), COVID-19 knowledge, perceived COVID-19 risk, subjective general health, and flu vaccine uptake as exogenous manifest regressors, and trust in science and epistemic trust as latent constructs in the model. Based on the results presented in Table 3, we report that perceived COVID-19 risk is by far the strongest predictor of vaccination attitudes followed by COVID-19 knowledge, and trust in science. We also report that respondents who regularly get vaccinated against the flu have more positive attitudes toward COVID-19 vaccination. Amongst the background variables, only gender has a significant effect on attitudes: male respondents (β_direct_ = 0.06, SE = 0.09) are slightly more positive towards the vaccines than females. We do not find a significant effect in the cases of epistemic trust and subjective general health on vaccination attitudes.

**Table 3:** Total effects on vaccine acceptance and vaccine attitudes

	Standardized coefficient	Bootstrapped S.E.
COVID-19 vaccine uptake		
COVID-19 vaccination attitudes	0.61*	0.01
Perceived behavioral control	-. 22*	0.01
Subjective norms	0.11*	0.01
Trust in science	0.23*	0.02
Epistemic trust	0.09*	0.01
COVID-19 knowledge	0.25*	0.04
Perceived COVID-19 risk	0.29*	0.01
Flu vaccine uptake	0.08*	0.01
COVID-19 vaccine attitudes		
Trust in science	0.38*	0.07
Epistemic trust	0.15*	0.05
COVID-19 knowledge	0.41*	0.19
Perceived COVID-19 risk	0.47*	0.04
Flu vaccine uptake	0.13*	0.05

Nr. of bootstrap samples: 1,000. Estimation method: diagonally weighted least squares (DWLS)

For the calculation of the total effects, see Additional file 5

* *p* < .05

### Trust in science

Finally, we drew paths from the background variables, COVID-19 knowledge, perceived COVID-19 risk, and epistemic trust towards trust in science. The model shows that COVID-19 knowledge is an important factor in increasing trust (β = 0.29, SE = 0.09). It is further revealed that individuals who perceive a higher risk of COVID-19 tend to trust science more (β = 0.39, SE = 0.03). Lining up with our expectations, we find a positive effect of epistemic trust on trust in science (β = 0.31, SE = 0.04). Last but not least, respondents with higher education and more income also trust science more. Age and gender have no effect on trust.

### Sub-group analyses

Previous research in Hungary has shown that socio-demographic characteristics were important determinants of attitudes towards vaccination [39, 40]. To test if our results hold in various sample sub-groups, we performed sub-group analyses taking into account age, gender, and the level of education. Full models are reported in Additional file 8; here we limit the discussion to the effects of trust in science and COVID-19 knowledge across the different levels of education (low, middle, upper).

Crucially, contrary to the groups of middle (β = 0.58, SE = 0.06) and upper education (β = 0.53, SME = 0.14), in the low-education sub-sample trust in science has no effect on COVID-19 vaccination attitudes (β = 0.01, SE = 0.31). Additionally, our findings show that knowledge has no effect on trust in science in the low-education sub-sample (β = 0.08, SE = 0.12). These figures reinforce that the effect of knowledge on vaccination attitudes is not mediated through trust. Nevertheless, the direct effect of knowledge on vaccination attitudes is still notable in this group (β = 0.64, SE = 0.12) indicating that increasing knowledge may strengthen pro-vaccination attitudes – and thus vaccine acceptance, and the mechanism is less complex than amongst better educated individuals where trust mediates the effect of knowledge. Interestingly, despite the less complex path from knowledge to vaccine acceptance in the low-educated group the total effect of knowledge is only slightly smaller (β_total_ = 0.14, SE_bootsrtap_ = 0.04) than in the case of people with university degree (β_total_ = 0.14, SE_bootsrtap_ = 0.05). We observe the lowest total effect of knowledge on vaccination among respondents with mid-level education (β_total_ = 0.08, SE_bootsrtap_ = 0.01).

## Discussion

In this study, we investigated the determinants of COVID-19 vaccine acceptance among Hungarian adults using the TPB theoretical framework. Consistent with most studies examining COVID-19 vaccination intentions, the results supported the hypothesis (H1) that the *attitude of individuals toward COVID-19 vaccination* is positively associated with vaccine acceptance. Also, attitudes toward vaccination were found to be the strongest predictor of COVID-19 vaccination as reported in the literature [41]. 

In line with our hypothesis (H2), *subjective norms* were also found to have a significant positive effect on COVID-19 vaccine acceptance, indicating that the more pressure an individual perceives from family and friends to get vaccinated, the more likely they are to accept the vaccine. This is consistent with the findings of previous studies [8, 16], whereas other studies found no significant relationship between these constructs [1, 7]. 

Contrary to our hypothesis (H3), *perceived behavioral control* has a significant but negative association with COVID-19 vaccine acceptance: the more control the person perceived over their intended behavior, the less they accepted the vaccine. This finding may be attributable to the timing of the Hungarian data collection, which took place relatively late into the pandemic, in November 2022. This represents an important difference from other studies employing the TPB to understand vaccination intentions [1, 7, 8]. The timing of data collection might have had two consequences. First, by that time, vaccines were widely available, so getting vaccinated no longer posed any difficulty (i.e., there was no perceived behavioral control). Second, with the availability of vaccines, the government allowed workplaces to make vaccination mandatory. Therefore, some individuals had no choice but to get vaccinated (or accepted dismissal). Meanwhile, an increasing number of people who were free to choose whether to get vaccinated decided against it, as reports of side effects and the spread of conspiracy theories became more common. Our findings indicate that the effect of perceived behavioral control depends strongly on the context. This aligns with the systematic literature review and meta-analysis by Limbu et al. [41] which highlight the differences between the TPB constructs in significance and strength across regions and study populations. This evidence underscores the importance of examining vaccine uptake in diverse contexts.

We extended the TPB model with three additional variables (trust in science, knowledge about COVID-19, and perceived risk of COVID-19) that we assumed to predict attitudes toward COVID-19 vaccination. Out of the three predictors, perceived risk of *COVID-19* had the strongest effect on attitudes toward COVID-19 vaccination. Consistent with our hypothesis (H7), the higher the individual’s perceived risk of COVID-19, the more positive their attitude toward vaccination. This result is consistent with previous studies [7, 42, 43], including two studies in Hungary [13, 40]. Furthermore, beyond the direct effect of perceived COVID-19 risk on vaccination attitude, we found an important indirect relationship mediated by trust in science.

Our results support the hypothesis that those who have more *knowledge about COVID-19* tend to have a more positive attitude toward COVID-19 vaccination (H6). This finding is in line with prior studies [1, 24, 25]. In addition, we showed that knowledge about COVID-19 is also indirectly related to attitudes toward vaccination through trust in science.

Consistent with our hypothesis (H4), *trust in science* is a strong predictor of attitudes toward COVID-19 vaccination. Those who trust scientists and healthcare professionals more tend to have more favorable attitudes towards vaccination, which means that trust indirectly increases vaccine uptake. This result is in line with earlier studies that have investigated the role of trust in COVID-19 vaccination both within [7, 8] or outside the theoretical framework of TPB [18–20]. 

Few studies have examined the factors influencing the development of individuals’ trust in science in the context of COVID-19 vaccination, and one contribution of our research is to try to fill this gap. To our knowledge, no previous work has explored the role of *epistemic trust* in the formation of trust in scientists and health care professionals. Our findings show that higher levels of epistemic trust are associated with higher levels of trust in science (H5), i.e. epistemic trust has an indirect positive effect on attitudes towards COVID-19 vaccination.

Our model also included the potential socioeconomic and health-related determinants of individuals’ attitudes toward COVID-19 vaccination. Of the socioeconomic variables (age, gender, education, income) only gender has a significant direct effect on vaccination attitudes. In line with most studies included in a recent systematic review [44], male respondents have slightly more favorable attitudes toward COVID-19 vaccination. In addition, our findings show that educational attainment and income have an indirect effect on vaccination attitude, mediated by trust in science.

As for health-related variables, consistent with the literature [1, 45], we found that past flu vaccination was a significant predictor of attitudes toward COVID-19 vaccination, i.e. those who regularly get vaccinated against flu are more likely to have a positive attitude toward COVID-19 vaccination. Self-reported health status in our study is not associated with respondents’ attitudes toward COVID-19 vaccination. This result is consistent with the findings of a systematic literature review by Adu et al. [44] However, another study reported a significant positive association between self-reported health status—measured by the presence of chronic conditions and self-reported health-related quality of life (HRQoL)—and the acceptance of COVID-19 vaccine [46]. These differing results may be attributable to variations in how self-reported health status was operationalized across studies.

As with most empirical studies, we must report a few limitations. First, since our study relied on an online sample, we can only carefully generalize our results to the whole population of Hungary. At the same time, there is evidence that such data produce similar results to population-based samples [47]. Second, although our sample was representative across key socio-demographic factors, vaccinated individuals were overrepresented. Hence, the attitudes toward COVID-19 vaccination are potentially more positive in our sample than in the population. Third, caution is advised when interpreting relationships as cause and effect. On the one hand, the study relied on cross-sectional data which is not ideal to uncover causal effects. On the other hand, the direction of causality may also be contested. Similar to other studies [48], we tested whether the level of perceived risk and knowledge affect trust in science. However, the opposite direction may also hold true. Individuals with greater trust in science are more likely to believe and pay closer attention to information provided by experts, and therefore, may possess more thorough knowledge about COVID-19—a line of reasoning also be found in the literature [21, 49]. Future studies should further explore the directionality of trust-mediated relationships between COVID-19-related knowledge, perceived COVID-19 risk, and vaccination attitude.

With regards to the generalizability of our findings, the theoretical logic underlying the extended TPB model – namely that trust in science, knowledge, and perceived risk influence vaccine acceptance indirectly through attitudes – could extend to vaccine hesitancy more generally. Prior research suggests that trust in science [50]and perceived risk [51, 52] are important antecedents of vaccination attitudes beyond the COVID-19 case. However, the strength and form of these relationships are likely to depend on contextual and disease-specific factors (e.g., perceived severity, novelty of the vaccine, politicization). We therefore view our findings as most directly relevant to COVID-19 vaccination, but potentially informative for understanding hesitancy toward other vaccines as well.

## Conclusions

The results of our study are consistent with previous research showing that COVID-19 vaccine uptake depends primarily on attitudes towards vaccination. Strong predictors of attitudes are perceived risk of COVID-19, knowledge about COVID-19, and trust in science. It is therefore important that communication to the public focuses on these interrelated factors in order to effectively manage COVID-19 and, presumably, similar pandemics. The more people trust science, the more accurately they understand the nature of the pandemic and the more seriously they take the health risks, the more open they will be to vaccination. Importantly, as knowledge and trust are interrelated, information campaigns cannot focus on only one or the other: knowledge without trust and vice versa are not enough to increase vaccine acceptance. However, as our sub-group analysis has revealed, low-educated people may be an exception; since trust in science has no effect on attitudes toward vaccination in this sup-group, increasing knowledge is key to fostering pro-vaccine attitudes. This may require alternative communication strategies. The complex, formal and overly technical vocabulary that has characterized scientific communication about COVID-19 vaccination in some countries [53] should be avoided in their case to promote text comprehension and engagement with scientific information. Since images convey complex information more effectively than text, especially for low-educated people, a short video that demonstrates the effectiveness of vaccines in an easily understandable and credible way can increase trust in vaccines and thus willingness to be vaccinated [54]. 

Our study also aimed to look at the determinants of trust in science in relation to COVID-19. The factors influencing trust in science may be important in the communication strategy for a future pandemic, too. Our results show that higher levels of epistemic trust, allowing individuals to acquire knowledge and integrate new information more effectively, higher levels of education, and higher income are associated with higher levels of trust in science. Since neither the level of epistemic trust, nor the level of educational attainment can be influenced in the short term, epidemiological communication strategies must be adapted to them. Clear and consistent communication [3] from trusted sources of information [55] are crucial to enhancing trust. As our sub-group analysis has shown, trust-building communication pays off particularly in the middle- and high-education groups. Combating false and misleading information plays a key role in strengthening trust in science. A variety of tools are already available for this purpose [56], ranging from fact-checking tools that assess the credibility of media sources and news to psychological inoculation strategies that build up resilience against misinformation about vaccines (e.g., the Bad Vaxx online game) [57]. These tools are not yet widely known, even though they would be useful in combating misinformation not only in relation to COVID-19 but in other areas as well.

## Supplementary Information

Additional file 1. Survey items in the study.

Additional file 2. Variables in the analysis and descriptive statistics.

Additional file 3. Measurement model.

Additional file 4. Structural model; Standard TPB and extended TPB comparison.

Additional file 5. The structural model with total effects on vaccination attitudes and vaccine uptake (bootstrapped standard errors).

Additional file 6. Inspection of missing data.

Additional file 7. Structural model (cat. ‘Not yet but I am planning to’ excluded).

Additional file 8. Sub-group analysis.

Additional file 9.

## Data Availability

The datasets generated and/or analyzed during the current study are available at [10.6084/m9.figshare.25112807.v1].

## Footnotes

1
https://ourworldindata.org/coronavirus; Date: 1 November 2022, Selected indicators: Confirmed cases, Confirmed deaths; Data source: World Health Organization; Accessed: 2 December 2025.

2
https://ourworldindata.org/covid-vaccinations; Accessed: 30 January, 2024.

3 National Sample HU; https://hu.nationalsample.com/; Accessed: 7 November 2025.

4 For the analysis of missing data see Additional file 6.

5 Based on the 2022 Census, the national data are as follows: Mean age: 49.9; gender: male: 47.4%, female: 52.6%; place of residence: Budapest: 18.1%, town: 52.8%, village: 29.1%; education: low: 20.0%, middle: 56.3%, high: 23.7%. https://nepszamlalas2022.ksh.hu/en/database/, Accessed: 5 November 2025.

6 As a robustness check, we run our main model excluding respondents in the 4th category (see Additional file 7).

7 Additionally, we report that the extended TPB provides a considerably better fit to the data than the standard TPB (Additional file 4; CFI = 0.838, RMSEA = 0.278).

## Abbreviations

COVID-19: Coronavirus desease 2019
TPB: Theory of Planned Behavior
SD: Standard Deviation
SEM: Structural Equation Modeling
CFI: Comparative Fit Index
RMSEA: Root Mean Square Error of Approximation
WLS: Weighted Least Squares
AVE: Average Variance Extracted
SE: Standard Error
