# Supplementary material for: Trust in science, knowledge and risk perception as predictors of COVID-19 vaccination: application of an extended Theory of Planned Behavior model in Hungary
Source: BMC Public Health. 2026 Feb 3;26:774. doi: 10.1186/s12889-026-26421-5 (PMC12955181; doi:10.1186/s12889-026-26421-5)
Supplement: Supplementary file 1 — Additional file 1. Survey items in the study. [file 12889_2026_26421_MOESM1_ESM.pdf]

## Survey items

| English | |
|---|---|
| *COVID-19 vaccine acceptance* | |
| Did you get vaccinated against Covid-19? | 1 = Yes and I plan to get more shots<br>2 = Yes and I don't plan to get more shots<br>3 = Not yet but I am planning to<br>4 = No and I don't plan to<br>888 = I am not sure<br>999 = I do not wish to answer |
| *COVID-19 vaccination attitudes* | |
| Covid-19 vaccines are effective in preventing infection and serious illness. | 1 = Strongly disagree<br><br>4 = Neither agree nor disagree<br>7 = Strongly agree<br>888 = I am not sure<br>999 = I do not wish to answer |
| Covid-19 vaccines are safe and their known side effects are negligible. | 1 = Strongly disagree<br><br>4 = Neither agree nor disagree<br>7 = Strongly agree<br>888 = I am not sure<br>999 = I do not wish to answer |
| By getting vaccinated, we take responsibility for others. | 1 = Strongly disagree<br><br>4 = Neither agree nor disagree<br>7 = Strongly agree<br>888 = I am not sure<br>999 = I do not wish to answer |
| *Perceived behavioral control* | |
| I could freely decide whether to get a Covid-19 vaccine. | 1 = I strongly disagree<br><br>5 = I strongly agree<br>888 = I am not sure<br>999 = I do not wish to answer |
| *Subjective norms* | |
| How acceptable is it in your family for someone not to get the Covid-19 vaccine even though they could? | 1 = Not acceptable at all<br><br>5 = Absolutely acceptable<br>888 = I am not sure<br>999 = I do not wish to answer |
| How acceptable is it in your circle of friends for someone not to get the Covid-19 vaccine even though they could? | 1 = Not acceptable at all<br><br>5 = Absolutely acceptable<br>888 = I am not sure<br>999 = I do not wish to answer |
| *Trust in science* | |
| How much do you trust scientists in general when it comes to epidemiological information? | 1 = I don't trust them at all<br><br>5 = I trust them a lot<br>888 = I am not sure<br>999 = I do not wish to answer |
| How much do you trust health professionals in general when it comes to epidemiological information? (medical doctors, epidemiological experts) | 1 = I don't trust them at all<br><br>5 = I trust them a lot<br>888 = I am not sure<br>999 = I do not wish to answer |
| How much do you trust your GP, doctor or pharmacist regarding epidemiological information? | 1 = I don't trust them at all<br><br>5 = I trust them a lot<br>888 = I am not sure<br>999 = I do not wish to answer |

| | |
|---|---|
| *Epistemic trust* | |
| I usually ask people for advice when I have a personal problem. | 1 = Strongly disagree<br><br>4 = Neither agree nor disagree<br>7 = Strongly agree<br>888 = I am not sure<br>999 = I do not wish to answer |
| I find information easier to trust and absorb when it comes from someone who knows me well. | 1 = Strongly disagree<br><br>4 = Neither agree nor disagree<br>7 = Strongly agree<br>888 = I am not sure<br>999 = I do not wish to answer |
| If I don't know what to do, my first instinct is to ask someone whose opinion I value. | 1 = Strongly disagree<br><br>4 = Neither agree nor disagree<br>7 = Strongly agree<br>888 = I am not sure<br>999 = I do not wish to answer |
| I often feel that people do not understand what I want and need. | 1 = Strongly disagree<br><br>4 = Neither agree nor disagree<br>7 = Strongly agree<br>888 = I am not sure<br>999 = I do not wish to answer |
| If you put too much faith in what people tell you, you are likely to get hurt. | 1 = Strongly disagree<br><br>4 = Neither agree nor disagree<br>7 = Strongly agree<br>888 = I am not sure<br>999 = I do not wish to answer |
| I don't usually act on advice that I get from others even when I think it's probably sound. | 1 = Stongly disagree<br><br>4 = Neither agree nor disagree<br>7 = Strongly agree<br>888 = I am not sure<br>999 = I do not wish to answer |
| *Perceived COVID-19 risk* | |
| The consequences of Covid-19 infection are severe. | 1 = I don't agree at all<br><br>5 = I totally agree<br>888 = I am not sure<br>999 = I do not wish to answer |
| If I were infected (again) by Covid-19, I would be hospitalized for sure. | 1 = I don't agree at all<br><br>5 = I totally agree<br>888 = I am not sure<br>999 = I do not wish to answer |
| *COVID-19 knowledge* | |
| Washing your hands thoroughly is an effective way to prevent Covid-19 infection. | 1 =True<br><br>2 = False<br>888 = I am not sure<br><br>999 = I do not wish to answer |
| Wearing masks reduces the spread of the coronavirus. | 1 = True<br><br>2 = False<br>888 = I am not sure<br><br>999 = I do not wish to answer |
| Antibiotics are effective against the Covid-19 disease. | 1 = True<br><br>2 = False<br>888 = I am not sure<br>999 = I do not wish to answer |

| | |
|---|---|
| Covid-19 vaccines can cause Covid-19 disease. | 1 = True<br>2 = False<br>888 = I am not sure<br>999 = I do not wish to answer |
| *Health block* | |
| How is your overall health? | 1 = Very good<br>2 = Good<br>3 = Satisfying<br>4 = Bad<br>5 = Very bad<br>888 = I am not sure<br>999 = I do not wish to answer |
| Do you usually get the flu shot? | 1 = Yes, regularly, each year<br>2 = Yes, from time to time<br>3 = I got it once or twice<br>4 = No, I have never got it<br>888 = I am not sure<br>999 = I do not wish to answer |
| *Socio-demographics* | |
| In which year were you born? | 999 = I do not wish to answer |
| What is your gender? | 0 = Male<br>1 = Female<br>999 = I do not wish to answer |
| What is your highest completed level of education? | 1 = Did not complete elementary school<br>2 = Elementary school<br>3 = Vocational training (vocational school, apprenticeship)<br>4 = High school diploma<br>5 = College degree (BA/BSc)<br>6 = University degree (MA/MSc)<br>7 = Doctoral degree (PhD/DLA) or higher<br>999 = I do not wish to answer |
| How do you manage financially... | 1 = I/we don't have any problems<br>2 = I/we are doing well if we budget<br>3 = I/we just get by on my/our monthly income<br>4 = I/we have financial problems each month<br>5 = I/we have our needs not met<br>888 = I am not sure<br>999 = I do not wish to answer |
