# Supplementary material for: Trust in science, knowledge and risk perception as predictors of COVID-19 vaccination: application of an extended Theory of Planned Behavior model in Hungary
Source: BMC Public Health. 2026 Feb 3;26:774. doi: 10.1186/s12889-026-26421-5 (PMC12955181; doi:10.1186/s12889-026-26421-5)
Supplement: Supplementary file 2 — Additional file 2. Variables in the analysis and descriptive statistics. [file 12889_2026_26421_MOESM2_ESM.pdf]

## Variables in the analysis and descriptive statistics

| | | Scale | N (%) | Mean (SD) | Note |
|---|---|---|---|---|---|
| **Manifest variables** | | | | | |
| COVID vaccine uptake | Did you get vaccinated against Covid-19? | Yes | 570 (74.9) | | 'Yes and I plan to get more shots'; (N = 189)<br>'Yes and I don't plan to get more shots'; (N = 373)<br>'Not yet but I am planning to' (N = 8) |
| | | No | 191 (25.1) | | 'No and I don't plan to' |
| Age | Age of the respondent calculated from the item 'In which year were you born?' | | | 48.8 (15.9) | In the analysis, age is scaled and centered. |
| Gender | What is your gender? | Male | 372 (48.8) | | |
| | | Female | 389 (51.2) | | |
| Education | What is your highest completed level of education? | Low | 121 (0.16) | | Did not complete elementary school; Elementary school |
| | | Middle | 474 (0.63) | | Vocational training; High school diploma |
| | | High | 166 (0.20) | | College or university degree |
| Subjective income | How do you manage financially? | 1 - 5 | | 3.22 (0.98) | Reversed from original scale; Higher values indicate more income |
| Subjective general health | How is your overall health? | 1-5 | | 3.56 (0.9) | Reversed from original scale; Higher values indicate a healthier respondent |
| Flu vaccine uptake | Do you usually get a flu vaccine? | 1-4 | | 1.8 (1.1) | Reversed from original scale; Higher values indicate larger vaccination propensity |
| Perceived behavioral control | I could freely decide whether to get a Covid-19 vaccine. | 1-5 | | 4.2 (1.3) | Larger values indicate more control. |
| **Additive indices** | | | | | |
| Subjective norms | - How acceptable is it in your family for someone not to get the Covid-19 vaccine even though they could?<br>- How acceptable is it in your circle of friends for someone not to get the Covid-19 vaccine even though they could? | 2-10 | | 5.09 (2.86) | Larger values indicate more pressure from friends and family. |
| COVID-19 knowledge | - Washing your hands thoroughly is an effective way to prevent Covid-19 infection.<br>- Wearing masks reduces the spread of the coronavirus.<br>- Antibiotics are effective against the Covid-19 disease.<br>- Covid-19 vaccines can cause Covid-19 disease. | 0-4 | | 2.79 (1.05) | Indicates the number of knowledge questions answered correctly. |
| Perceived COVID-19 risk | - The consequences of Covid-19 infection are severe.<br>- If I were infected (again) by Covid-19, I would be hospitalized for sure. | 2-10 | | 6.05 (2.17) | Larger values indicate more perceived risk. |

| | | | | |
|---|---|---|---|---|
| **Latent factors** | | | | |
| COVID-19 vaccine attitudes | Covid-19 vaccines are effective in preventing infection and serious illness. | 1-7 | 3.25 (1.49) | Larger values indicate stronger agreement with the statement. |
| | Covid-19 vaccines are safe and their known side effects are negligible. | 1-7 | 2.77 (1.45) | Larger values indicate stronger agreement with the statement. |
| | By getting vaccinated, we take responsibility for others. | 1-7 | 3.37 (1.52) | Larger values indicate stronger agreement with the statement. |
| Trust in science | How much do you trust scientists in general when it comes to epidemiological information? | 1-7 | 3.41 (1.28) | Larger values indicate more trust. |
| | How much do you trust health professionals in general when it comes to epidemiological information? (medical doctors, epidemiological experts) | 1-7 | 3.40 (1.23) | Larger values indicate more trust. |
| | How much do you trust your GP, doctor or pharmacist regarding epidemiological information? | 1-7 | 3.58 (1.24) | Larger values indicate more trust. |
| Epistemic trust | I usually ask people for advice when I have a personal problem. | 1-7 | 3.95 (1.72) | Larger values indicate stronger agreement with the statement. |
| | I find information easier to trust and absorb when it comes from someone who knows me well. | 1-7 | 4.99 (1.61) | Larger values indicate stronger agreement with the statement. |
| | If I don't know what to do, my first instinct is to ask someone whose opinion I value. | 1-7 | 4.59 (1.76) | Larger values indicate stronger agreement with the statement. |
