# Supplementary material for: Trust in science, knowledge and risk perception as predictors of COVID-19 vaccination: application of an extended Theory of Planned Behavior model in Hungary
Source: BMC Public Health. 2026 Feb 3;26:774. doi: 10.1186/s12889-026-26421-5 (PMC12955181; doi:10.1186/s12889-026-26421-5)
Supplement: Supplementary file 3 — Additional file 3. Measurement model. [file 12889_2026_26421_MOESM3_ESM.pdf]

Measurement model

```
m <- '
   # Measurement model
         t =~ t1 + t2 + t3
         attitude =~ att1 + att2 + att3
         et =~ et1 + et2 + et3
      '

cfa <- lavaan::cfa(m, data=data)
summary(cfa, fit.measures = T, standardized = T)
```

```
lavaan 0.6.18.1972 ended normally after 44 iterations

  Estimator                                         ML
  Optimization method                           NLMINB
  Number of model parameters                        21

  Number of observations                           996

Model Test User Model:

  Test statistic                                73.354
  Degrees of freedom                                24
  P-value (Chi-square)                           0.000

Model Test Baseline Model:

  Test statistic                              5452.701
  Degrees of freedom                                36
  P-value                                        0.000

User Model versus Baseline Model:

  Comparative Fit Index (CFI)                    0.991
  Tucker-Lewis Index (TLI)                       0.986

Loglikelihood and Information Criteria:

  Loglikelihood user model (H0)             -13294.945
  Loglikelihood unrestricted model (H1)             NA

  Akaike (AIC)                               26631.891
  Bayesian (BIC)                             26734.870
  Sample-size adjusted Bayesian (SABIC)      26668.173

Root Mean Square Error of Approximation:

  RMSEA                                          0.045
  90 Percent confidence interval - lower         0.034
  90 Percent confidence interval - upper         0.057
  P-value H_0: RMSEA <= 0.050                    0.719
  P-value H_0: RMSEA >= 0.080                    0.000

Standardized Root Mean Square Residual:

  SRMR                                           0.024

Parameter Estimates:

  Standard errors                             Standard
  Information                                 Expected
  Information saturated (h1) model          Structured

Latent Variables:
                   Estimate  Std.Err  z-value  P(>|z|)   Std.lv  Std.all
  t =~
    t1                1.000                               1.029    0.841
    t2                1.090    0.030   36.459    0.000    1.122    0.941
```

```
    t3                0.878    0.032   27.262    0.000    0.904    0.747
  attitude =~
    att1              1.000                               1.348    0.921
    att2              0.885    0.024   36.384    0.000    1.192    0.831
    att3              0.993    0.024   41.270    0.000    1.338    0.886
  et =~
    et1               1.000                               1.167    0.688
    et2               1.011    0.054   18.777    0.000    1.179    0.744
    et3               1.238    0.066   18.853    0.000    1.444    0.818

Covariances:
                   Estimate  Std.Err  z-value  P(>|z|)   Std.lv  Std.all
  t ~~
    attitude          1.045    0.063   16.550    0.000    0.753    0.753
    et                0.346    0.048    7.235    0.000    0.288    0.288
  attitude ~~
    et                0.256    0.059    4.320    0.000    0.163    0.163

Variances:
                   Estimate  Std.Err  z-value  P(>|z|)   Std.lv  Std.all
   .t1                0.439    0.026   16.711    0.000    0.439    0.293
   .t2                0.162    0.021    7.740    0.000    0.162    0.114
   .t3                0.648    0.033   19.753    0.000    0.648    0.442
   .att1              0.323    0.028   11.513    0.000    0.323    0.151
   .att2              0.639    0.035   18.092    0.000    0.639    0.310
   .att3              0.488    0.033   14.929    0.000    0.488    0.214
   .et1               1.516    0.089   17.072    0.000    1.516    0.527
   .et2               1.118    0.076   14.630    0.000    1.118    0.446
   .et3               1.028    0.097   10.559    0.000    1.028    0.330
    t                 1.060    0.067   15.923    0.000    1.000    1.000
    attitude          1.817    0.098   18.578    0.000    1.000    1.000
    et                1.361    0.124   11.011    0.000    1.000    1.000
```

Reliability

```
semTools::reliability(cfa,return.total=TRUE)
```

```
               t  attitude        et     total
alpha  0.8829051 0.9081355 0.7975309 0.8388271
omega  0.8888684 0.9100087 0.8009998 0.9173399
omega2 0.8888684 0.9100087 0.8009998 0.9173399
omega3 0.8924351 0.9103073 0.8016170 0.9137356
avevar 0.7287837 0.7718546 0.5747425 0.6763242
```

Discriminant validity

```
discriminantValidity(cfa, cutoff = 0.9, merge = FALSE, level = 0.95)
```

```
       lhs op      rhs       est   ci.lower  ci.upper Df      AIC      BIC    Chisq Chisq
diff
1        t ~~ attitude 0.7602859 0.72355364 0.7970181 25 20613.45 20706.15 155.3441
98.28152
2        t ~~       et 0.2951267 0.21922186 0.3710316 25 21092.74 21185.44 634.6342
577.57163
3 attitude ~~       et 0.1747337 0.09445467 0.2550127 25 21178.52 21271.21 720.4066
663.34410

      RMSEA Df diff    Pr(>Chisq)
1 0.3575385       1   3.629366e-23
2 0.8704309       1 1.265617e-127
3 0.9329310       1 2.799472e-146
```
