# Supplementary material for: Trust in science, knowledge and risk perception as predictors of COVID-19 vaccination: application of an extended Theory of Planned Behavior model in Hungary
Source: BMC Public Health. 2026 Feb 3;26:774. doi: 10.1186/s12889-026-26421-5 (PMC12955181; doi:10.1186/s12889-026-26421-5)
Supplement: Supplementary file 4 — Additional file 4. Structural model; Standard TPB and extended TPB comparison. [file 12889_2026_26421_MOESM4_ESM.pdf]

Structural model

```
m <- '
   # Measurement model
         t =~ t1 + t2 + t3
         attitude =~ att1 + att2 + att3
         et =~ et1 + et2 + et3

   # Regression models
      vaccinated ~ attitude + control + subjectivenorms
      attitude ~ age_sc + gender + edu_low + edu_middle + income +
                  health + fluvaccine + risk +
                  t + knowledge + et
      t ~ age_sc + gender + edu_low + edu_middle + income +
         et + risk + knowledge
      '
model <- lavaan::sem(m, data=data, estimator = "WLSMV")
summary(model, fit.measures = T, standardized = T)
```

```
lavaan 0.6.18.1972 ended normally after 111 iterations

  Estimator                                         DWLS
  Optimization method                             NLMINB
  Number of model parameters                         107

  Number of observations                             761

Model Test User Model:
                                              Standard      Scaled
  Test Statistic                               239.624     318.025
  Degrees of freedom                               124         124
  P-value (Chi-square)                           0.000       0.000
  Scaling correction factor                                  0.923
  Shift parameter                                           58.451
    simple second-order correction

Model Test Baseline Model:

  Test statistic                             11273.648    4595.442
  Degrees of freedom                               155         155
  P-value                                        0.000       0.000
  Scaling correction factor                                  2.504

User Model versus Baseline Model:

  Comparative Fit Index (CFI)                    0.990       0.956
  Tucker-Lewis Index (TLI)                       0.987       0.945

  Robust Comparative Fit Index (CFI)                         0.990
  Robust Tucker-Lewis Index (TLI)                            0.987

Root Mean Square Error of Approximation:

  RMSEA                                          0.035       0.045
  90 Percent confidence interval - lower         0.028       0.039
  90 Percent confidence interval - upper         0.042       0.052
  P-value H_0: RMSEA <= 0.050                    1.000       0.889
  P-value H_0: RMSEA >= 0.080                    0.000       0.000

  Robust RMSEA                                               0.044
  90 Percent confidence interval - lower                     0.038
  90 Percent confidence interval - upper                     0.050
  P-value H_0: Robust RMSEA <= 0.050                         0.962
  P-value H_0: Robust RMSEA >= 0.080                         0.000

Standardized Root Mean Square Residual:

  SRMR                                           0.039       0.039
```

```
Parameter Estimates:

  Standard errors                             Robust.sem
  Information                                   Expected
  Information saturated (h1) model          Unstructured

Latent Variables:
                   Estimate  Std.Err  z-value  P(>|z|)   Std.lv  Std.all
  t =~
    t1                1.000                               1.109    0.868
    t2                1.036    0.030   34.828    0.000    1.149    0.933
    t3                0.830    0.038   21.913    0.000    0.921    0.743
  attitude =~
    att1              1.000                               1.384    0.928
    att2              0.847    0.023   36.753    0.000    1.172    0.806
    att3              0.987    0.020   48.940    0.000    1.366    0.901
  et =~
    et1               1.000                               1.229    0.714
    et2               1.028    0.089   11.600    0.000    1.263    0.784
    et3               1.097    0.085   12.881    0.000    1.348    0.764

Regressions:
                   Estimate  Std.Err  z-value  P(>|z|)   Std.lv  Std.all
  vaccinated ~
    attitude          0.191    0.011   17.127    0.000    0.265    0.611
    control          -0.073    0.010   -7.300    0.000   -0.073   -0.218
    subjectivenrms    0.016    0.005    3.377    0.001    0.016    0.107
  attitude ~
    age_sc           -0.072    0.038   -1.888    0.059   -0.052   -0.051
    gender            0.182    0.066    2.751    0.006    0.132    0.066
    edu_low          -0.082    0.119   -0.691    0.489   -0.059   -0.022
    edu_middle       -0.105    0.096   -1.095    0.273   -0.076   -0.037
    income            0.002    0.036    0.053    0.958    0.001    0.001
    health            0.026    0.038    0.701    0.483    0.019    0.017
    fluvaccine        0.168    0.031    5.357    0.000    0.121    0.130
    risk              0.208    0.025    8.193    0.000    0.151    0.322
    t                 0.473    0.067    7.035    0.000    0.379    0.379
    knowledge         0.389    0.044    8.806    0.000    0.281    0.294
    et                0.039    0.049    0.801    0.423    0.035    0.035
  t ~
    age_sc            0.061    0.042    1.455    0.146    0.055    0.054
    gender           -0.027    0.071   -0.380    0.704   -0.024   -0.012
    edu_low          -0.390    0.127   -3.073    0.002   -0.352   -0.128
    edu_middle       -0.390    0.088   -4.442    0.000   -0.352   -0.170
    income            0.159    0.038    4.223    0.000    0.143    0.140
    et                0.277    0.047    5.924    0.000    0.306    0.306
    risk              0.204    0.019   10.837    0.000    0.184    0.393
    knowledge         0.315    0.040    7.807    0.000    0.284    0.297

Covariances:
                     Estimate  Std.Err  z-value  P(>|z|)   Std.lv  Std.all
  control ~~
    subjectivenrms     -0.022    0.138   -0.157    0.875   -0.022   -0.006
    age_sc              0.306    0.042    7.249    0.000    0.306    0.240
    gender              0.028    0.024    1.180    0.238    0.028    0.043
    edu_low             0.009    0.016    0.538    0.591    0.009    0.018
    edu_middle         -0.059    0.022   -2.696    0.007   -0.059   -0.094
    income              0.026    0.049    0.540    0.589    0.026    0.021
    health              0.056    0.047    1.203    0.229    0.056    0.048
    fluvaccine          0.075    0.045    1.659    0.097    0.075    0.054
    risk                0.731    0.114    6.426    0.000    0.731    0.265
    knowledge           0.181    0.051    3.540    0.000    0.181    0.134
  subjectivenorms ~~
    age_sc              0.821    0.102    8.047    0.000    0.821    0.292
    gender              0.188    0.051    3.670    0.000    0.188    0.132
```

```
    edu_low           -0.006    0.039   -0.151    0.880   -0.006   -0.006
    edu_middle        -0.133    0.050   -2.638    0.008   -0.133   -0.096
    income             0.077    0.108    0.713    0.476    0.077    0.028
    health            -0.300    0.093   -3.218    0.001   -0.300   -0.116
    fluvaccine         1.096    0.122    9.001    0.000    1.096    0.357
    risk               2.457    0.258    9.531    0.000    2.457    0.402
    knowledge          1.281    0.106   12.049    0.000    1.281    0.428
  age_sc ~~
    gender             0.133    0.017    7.707    0.000    0.133    0.269
    edu_low            0.017    0.014    1.220    0.222    0.017    0.049
    edu_middle        -0.059    0.018   -3.357    0.001   -0.059   -0.125
    income            -0.006    0.033   -0.194    0.846   -0.006   -0.007
    health            -0.185    0.031   -5.924    0.000   -0.185   -0.207
    fluvaccine         0.408    0.040   10.171    0.000    0.408    0.386
    risk               0.615    0.074    8.315    0.000    0.615    0.292
    knowledge          0.398    0.036   10.971    0.000    0.398    0.385
  gender ~~
    edu_low           -0.003    0.007   -0.426    0.670   -0.003   -0.016
    edu_middle        -0.018    0.009   -2.047    0.041   -0.018   -0.074
    income             0.034    0.018    1.898    0.058    0.034    0.069
    health            -0.003    0.016   -0.213    0.832   -0.003   -0.008
    fluvaccine         0.082    0.019    4.256    0.000    0.082    0.153
    risk               0.064    0.039    1.616    0.106    0.064    0.059
    knowledge          0.065    0.019    3.410    0.001    0.065    0.124
  edu_low ~~
    edu_middle        -0.101    0.007  -14.293    0.000   -0.101   -0.574
    income            -0.040    0.014   -2.841    0.005   -0.040   -0.111
    health            -0.020    0.012   -1.678    0.093   -0.020   -0.062
    fluvaccine        -0.008    0.014   -0.585    0.559   -0.008   -0.022
    risk               0.024    0.032    0.736    0.462    0.024    0.031
    knowledge         -0.039    0.014   -2.680    0.007   -0.039   -0.101
  edu_middle ~~
    income            -0.036    0.017   -2.080    0.038   -0.036   -0.076
    health            -0.059    0.016   -3.783    0.000   -0.059   -0.135
    fluvaccine        -0.046    0.019   -2.366    0.018   -0.046   -0.088
    risk              -0.026    0.038   -0.679    0.497   -0.026   -0.025
    knowledge         -0.068    0.019   -3.634    0.000   -0.068   -0.133
  income ~~
    health             0.258    0.036    7.096    0.000    0.258    0.292
    fluvaccine         0.026    0.039    0.673    0.501    0.026    0.025
    risk               0.122    0.082    1.484    0.138    0.122    0.058
    knowledge          0.209    0.037    5.693    0.000    0.209    0.205
  health ~~
    fluvaccine        -0.182    0.035   -5.196    0.000   -0.182   -0.187
    risk              -0.348    0.081   -4.292    0.000   -0.348   -0.180
    knowledge         -0.082    0.036   -2.315    0.021   -0.082   -0.087
  fluvaccine ~~
    risk               0.828    0.086    9.617    0.000    0.828    0.361
    knowledge          0.319    0.040    7.893    0.000    0.319    0.284
  risk ~~
    knowledge          0.882    0.083   10.568    0.000    0.882    0.394

Variances:
                   Estimate  Std.Err  z-value  P(>|z|)   Std.lv  Std.all
   .t1                0.404    0.051    7.888    0.000    0.404    0.247
   .t2                0.197    0.035    5.578    0.000    0.197    0.130
   .t3                0.689    0.058   11.908    0.000    0.689    0.448
   .att1              0.309    0.045    6.884    0.000    0.309    0.139
   .att2              0.741    0.062   11.951    0.000    0.741    0.350
   .att3              0.434    0.054    7.965    0.000    0.434    0.189
   .et1               1.450    0.144   10.091    0.000    1.450    0.490
   .et2               0.999    0.153    6.538    0.000    0.999    0.385
   .et3               1.293    0.155    8.318    0.000    1.293    0.416
   .vaccinated        0.106    0.006   17.619    0.000    0.106    0.564
   .t                 0.556    0.064    8.682    0.000    0.452    0.452
   .attitude          0.471    0.049    9.593    0.000    0.246    0.246
```

```
    et                   1.510    0.175     8.645    0.000    1.000    1.000
    control              1.668    0.103    16.156    0.000    1.668    1.000
    subjectivenrms       8.160    0.271    30.150    0.000    8.160    1.000
    age_sc               0.972    0.033    29.547    0.000    0.972    1.000
    gender               0.250    0.000   617.690    0.000    0.250    1.000
    edu_low              0.132    0.009    14.594    0.000    0.132    1.000
    edu_middle           0.235    0.004    54.330    0.000    0.235    1.000
    income               0.956    0.048    19.898    0.000    0.956    1.000
    health               0.818    0.042    19.324    0.000    0.818    1.000
    fluvaccine           1.153    0.054    21.252    0.000    1.153    1.000
    risk                 4.574    0.215    21.284    0.000    4.574    1.000
    knowledge            1.096    0.045    24.436    0.000    1.096    1.000
```

Power analysis

```
ph <- semPower(type = 'post-hoc', 0.045, "RMSEA", alpha = 0.05, N = 761, df = 124)
summary(ph)
```

```
semPower: Post hoc power analysis

 F0                         0.251100
 RMSEA                      0.045000
 Mc                         0.882012

 df                         124
 Num Observations           761
 NCP                        190.8360

 Critical Chi-Square        150.9894
 Alpha                      0.050000
 Beta                       2.304987e-10
 Power (1 - Beta)           > 0.9999
 Implied Alpha/Beta Ratio 2.169210e+08
```

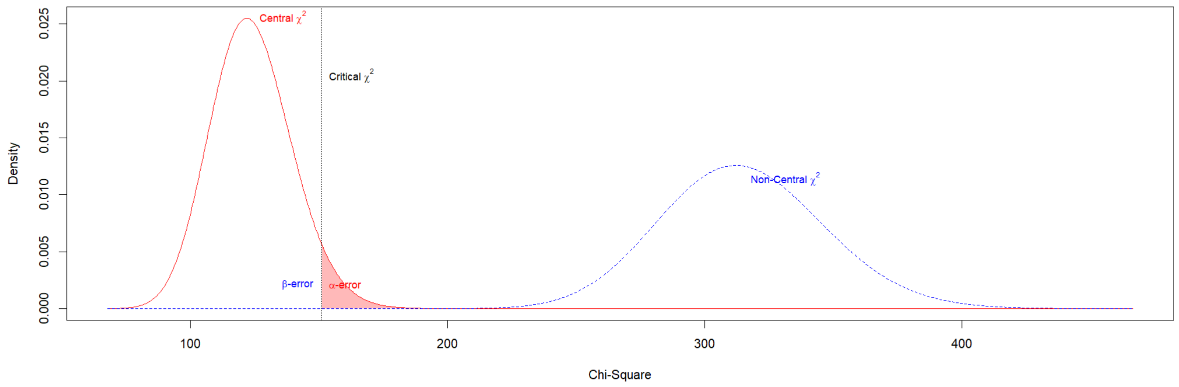

```
semPower.powerPlot.byEffect('RMSEA', alpha = 0.05, N = 761, df = 124,
                            effect.min = 0.001, effect.max = 0.1)
```

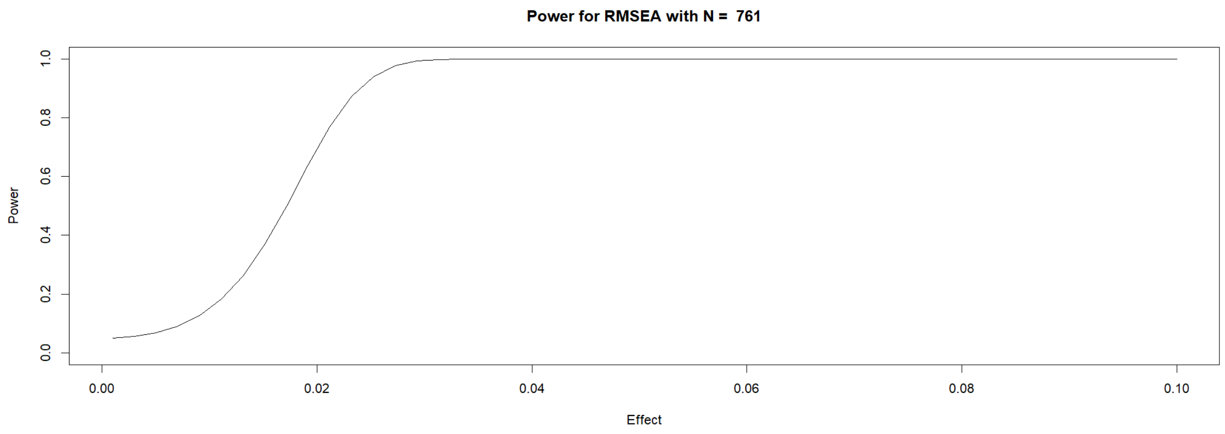

The standard TPB model

```
m <- '
   # Measurement model
         attitude =~ att1 + att2 + att3

   # Regression models
      vaccinated ~ attitude + control + subjectivenorms
      '
model <- lavaan::sem(m, data=data, estimator = "WLSMV")
summary(model, fit.measures = T, standardized = T)
```

```
  Estimator                                         DWLS
  Optimization method                             NLMINB
  Number of model parameters                          13

  Number of observations                             761

Model Test User Model:
                                              Standard      Scaled
  Test Statistic                               479.283     372.349
  Degrees of freedom                                 8           8
  P-value (Unknown)                                 NA       0.000
  Scaling correction factor                                  1.298
  Shift parameter                                            3.124
    simple second-order correction

Model Test Baseline Model:

  Test statistic                              2915.999    1713.758
  Degrees of freedom                                14          14
  P-value                                           NA       0.000
  Scaling correction factor                                  1.707

User Model versus Baseline Model:

  Comparative Fit Index (CFI)                    0.838       0.786
  Tucker-Lewis Index (TLI)                       0.716       0.625

  Robust Comparative Fit Index (CFI)                         0.838
  Robust Tucker-Lewis Index (TLI)                            0.716

Root Mean Square Error of Approximation:

  RMSEA                                          0.278       0.245
  90 Percent confidence interval - lower         0.258       0.224
  90 Percent confidence interval - upper         0.300       0.266
  P-value H_0: RMSEA <= 0.050                    0.000       0.000
  P-value H_0: RMSEA >= 0.080                    1.000       1.000

  Robust RMSEA                                               0.279
  90 Percent confidence interval - lower                     0.255
  90 Percent confidence interval - upper                     0.303
  P-value H_0: Robust RMSEA <= 0.050                         0.000
  P-value H_0: Robust RMSEA >= 0.080                         1.000

Standardized Root Mean Square Residual:

  SRMR                                           0.177       0.177

Parameter Estimates:

  Standard errors                           Robust.sem
  Information                                 Expected
  Information saturated (h1) model        Unstructured
```

```
Latent Variables:
                   Estimate  Std.Err  z-value  P(>|z|)   Std.lv  Std.all
  attitude =~
    att1              1.000                               1.401    0.939
    att2              0.841    0.024   34.403    0.000    1.179    0.810
    att3              0.952    0.025   38.686    0.000    1.334    0.880

Regressions:
                   Estimate  Std.Err  z-value  P(>|z|)   Std.lv  Std.all
  vaccinated ~
    attitude          0.192    0.010   19.767    0.000    0.269    0.620
    control          -0.036    0.011   -3.148    0.002   -0.036   -0.107
    subjectivenrms    0.049    0.005    9.510    0.000    0.049    0.322

Covariances:
                   Estimate  Std.Err  z-value  P(>|z|)   Std.lv  Std.all
  control ~~
    subjectivenrms   -0.055    0.138   -0.399    0.690   -0.055   -0.015

Variances:
                   Estimate  Std.Err  z-value  P(>|z|)   Std.lv  Std.all
   .att1              0.262    0.044    5.893    0.000    0.262    0.118
   .att2              0.727    0.067   10.884    0.000    0.727    0.344
   .att3              0.518    0.063    8.168    0.000    0.518    0.226
   .vaccinated        0.094    0.008   11.945    0.000    0.094    0.499
    attitude          1.962    0.080   24.420    0.000    1.000    1.000
    control           1.655    0.103   16.054    0.000    1.655    1.000
    subjectivenrms    8.194    0.271   30.237    0.000    8.194    1.000
```

The standard vs. the extended TPB model

Measures of fit	Cut-off value	Standard TPB	Extended TPB
Comparative Fit Index (CFI)	> 0.95	0.838	0.990
Tucker-Lewis Index (TLI)	> 0.95	0.716	0.987
Root Mean Square Error of Approximation (RMSEA)	< 0.06	0.278	0.035
Standardized Root Mean Square Residual (SRMR)	< 0.06	0.177	0.039

TPB variables	Standard TPB Estimate (S.E.)	Extended TPB Estimate (S.E.)
Vaccination attitudes	0.192 (0.010)	0.191 (0.011)
Perceived behavioral control	–0.036 (0.011)	–0.073 (0.010)
Subjective norms	0.049 (0.005)	0.016 (0.005)
