# Supplementary material for: Trust in science, knowledge and risk perception as predictors of COVID-19 vaccination: application of an extended Theory of Planned Behavior model in Hungary
Source: BMC Public Health. 2026 Feb 3;26:774. doi: 10.1186/s12889-026-26421-5 (PMC12955181; doi:10.1186/s12889-026-26421-5)
Supplement: Supplementary file 5 — Additional file 5. The structural model with total effects on vaccination attitudes and vaccine uptake (bootstrapped standard errors). [file 12889_2026_26421_MOESM5_ESM.pdf]

The structural model with total effects on vaccination attitudes and vaccine uptake (bootstrapped standard errors)

Trust in science

```
m <- '
   # Measurement model
         t =~ t1 + t2 + t3
         attitude =~ att1 + att2 + att3
         et =~ et1 + et2 + et3

   # Regression models
      vaccinated ~ b*attitude + control + subjectivenorms
      attitude ~ age_sc + gender + edu_low + edu_middle + income +
                  health + fluvaccine + risk +
                  a*t + knowledge + et
      t ~ age_sc + gender + edu_low + edu_middle + income +
         et + risk + knowledge

   # Path from trust in science to vaccination
      eff_1:= a*b
      '

model <- lavaan::sem(m, data=data, estimator = "DWLS", se = "bootstrap",
                     bootstrap = 1000,
                     test="scaled.shifted", verbose = T)
summary(model, fit.measures = T, standardized = T)
```

```
Defined Parameters:
                   Estimate  Std.Err  z-value  P(>|z|)   Std.lv  Std.all
    eff_1             0.091    0.015    5.870    0.000    0.101    0.232
```

Epistemic trust

```
m <- '
   # Measurement model
         t =~ t1 + t2 + t3
         attitude =~ att1 + att2 + att3
         et =~ et1 + et2 + et3

   # Regression models
      vaccinated ~ c*attitude + control + subjectivenorms
      attitude ~ age_sc + gender + edu_low + edu_middle + income +
                  health + fluvaccine + risk +
                  b*t + knowledge + d*et
      t ~ age_sc + gender + edu_low + edu_middle + income +
         a*et + risk + knowledge

   # Path from epistemic trust to attitudes
      eff_1:= d + a*b
   # Path from epistemic trust to vaccination
      eff_2:= d*c + a*b*c
      '

model <- lavaan::sem(m, data=data, estimator = "DWLS", se = "bootstrap",
                     bootstrap = 1000,
                     test="scaled.shifted", verbose = T)
summary(model, fit.measures = T, standardized = T)
```

```
Defined Parameters:
                   Estimate  Std.Err  z-value  P(>|z|)   Std.lv  Std.all
    eff_1             0.170    0.051    3.369    0.001    0.151    0.151
    eff_2             0.033    0.009    3.436    0.001    0.040    0.092
```

COVID-19-related knowledge

```
m <- '
   # Measurement model
         t =~ t1 + t2 + t3
         attitude =~ att1 + att2 + att3
         et =~ et1 + et2 + et3

   # Regression models
      vaccinated ~ d*attitude + control + subjectivenorms
      attitude ~ age_sc + gender + edu_low + edu_middle + income +
                  health + fluvaccine + risk +
                  c*t + b*knowledge + et
      t ~ age_sc + gender + edu_low + edu_middle + income +
         et + risk + a*knowledge

   # Path from knowledge to attitudes
      eff_1:= a*c + b
   # Path from knowledge to vaccination
      eff_2:= b*d + a*c*d
      '

model <- lavaan::sem(m, data=data, estimator = "DWLS", se = "bootstrap",
                     bootstrap = 1000,
                     test="scaled.shifted", verbose = T)
summary(model, fit.measures = T, standardized = T)
```

```
Defined Parameters:
                   Estimate  Std.Err  z-value  P(>|z|)   Std.lv  Std.all
    eff_1             0.538    0.190    2.827    0.005    0.388    0.407
    eff_2             0.103    0.038    2.687    0.007    0.103    0.248
```

Perceived COVID-19 risk

```
m <- '
   # Measurement model
         t =~ t1 + t2 + t3
         attitude =~ att1 + att2 + att3
         et =~ et1 + et2 + et3

   # Regression models
      vaccinated ~ c*attitude + control + subjectivenorms
      attitude ~ age_sc + gender + edu_low + edu_middle + income +
                  health + fluvaccine + d*risk +
                  b*t + knowledge + et
      t ~ age_sc + gender + edu_low + edu_middle + income +
         et + a*risk + knowledge

   # Path from risk to attitudes
      eff_1:= d + a*b
   # Path from risk to vaccination
      eff_2:= c*d + a*b*c
      '

model <- lavaan::sem(m, data=data, estimator = "DWLS", se = "bootstrap",
                     bootstrap = 1000,
                     test="scaled.shifted", verbose = T)
summary(model, fit.measures = T, standardized = T)
```

```
Defined Parameters:
                   Estimate  Std.Err  z-value  P(>|z|)   Std.lv  Std.all
    eff_1             0.305    0.040    7.623    0.000    0.220    0.471
    eff_2             0.058    0.009    6.357    0.000    0.058    0.288
```

Flu vaccine uptake

```
# FLU VACCINE UPTAKE
m <- '
   # Measurement model
         t =~ t1 + t2 + t3
         attitude =~ att1 + att2 + att3
         et =~ et1 + et2 + et3

   # Regression models
      vaccinated ~ b*attitude + control + subjectivenorms
      attitude ~ age_sc + gender + edu_low + edu_middle + income +
                  health + a*fluvaccine + risk +
                  t  + knowledge + et
      t ~ age_sc + gender + edu_low + edu_middle + income +
         et + risk + knowledge

   # Path from flu vaccination to vaccination
      eff_1:= a*b
      '

model <- lavaan::sem(m, data=data, estimator = "DWLS", se = "bootstrap",
                     bootstrap = 1000,
                     test="scaled.shifted", verbose = T)
summary(model, fit.measures = T, standardized = T)
```

```
Defined Parameters:
                   Estimate  Std.Err  z-value  P(>|z|)   Std.lv  Std.all
    eff_1             0.032    0.009    3.512    0.000    0.032    0.080
```
