# Supplementary material for: Trust in science, knowledge and risk perception as predictors of COVID-19 vaccination: application of an extended Theory of Planned Behavior model in Hungary
Source: BMC Public Health. 2026 Feb 3;26:774. doi: 10.1186/s12889-026-26421-5 (PMC12955181; doi:10.1186/s12889-026-26421-5)
Supplement: Supplementary file 6 — Additional file 6. Inspection of missing data. [file 12889_2026_26421_MOESM6_ESM.pdf]

Inspection of missing data

As explained in the main text of the paper, our sample size shrinks to 761 due to the list-wise deletion of cases. During our investigation into missing records, we find that there are four variables in our analysis that have particularly high missing values: COVID-19 related knowledge, Perceived COVID-19 risk, Subjective norms, and ATT2 (the second component of the latent factor Attitudes toward vaccination). The MCAR test indicates that data is not completely missing at random (rejecting the null-hypothesis). To find the structure in the missing data, and particularly in the cases of the four most problematic variables, we estimated four binary logit models with the dependent variables at 1 when the record is missing and 0 otherwise. We included age, gender, income, education, subjective general health, and place of residence as regressors – variables with only a few missing values. Model results show, that, first, male respondents were more likely to fill in the knowledge bloc of the questionnaire. Second, older respondents were more likely to avoid the questions that compose the risk variable than younger respondents. This effect, however, while statistically significant, is marginal in size. Third, individuals with the highest education level were more likely to respond to the subjective norms questions, as well as to the ATT2 item.

Number of missing records

```
full %>% ff_glimpse
```

```
$Continuous
                          label var_type    n missing_n missing_percent     mean     sd
t1                           t1    <dbl> 1435        65             4.3      3.4    1.2
t2                           t2    <dbl> 1433        67             4.5      3.4    1.2
t3                           t3    <dbl> 1432        68             4.5      3.6    1.2
att1                       att1    <dbl> 1402        98             6.5      3.2    1.5
att2                       att2    <dbl> 1391       109             7.3      2.7    1.4
att3                       att3    <dbl> 1413        87             5.8      3.3    1.5
et1                         et1    <dbl> 1465        35             2.3      3.8    1.7
et2                         et2    <dbl> 1461        39             2.6      4.9    1.6
et3                         et3    <dbl> 1460        40             2.7      4.5    1.8
control                 control    <dbl> 1445        55             3.7      4.3    1.3
subjectivenorms subjectivenorms    <dbl> 1361       139             9.3      5.0    2.8
age                         age    <dbl> 1500         0             0.0     49.1   16.2
income                   income    <dbl> 1484        16             1.1      3.2    1.0
health                   health    <dbl> 1487        13             0.9      3.5    0.9
fluvaccine           fluvaccine    <dbl> 1469        31             2.1      1.8    1.1
risk                       risk    <dbl> 1144       356            23.7      6.0    2.2
knowledge             knowledge    <dbl>  916       584            38.9      2.8    1.1
ID                           ID    <dbl> 1500         0             0.0 364542.4 1924.7
```

Missing values map

```
full %>% missing_plot()
```

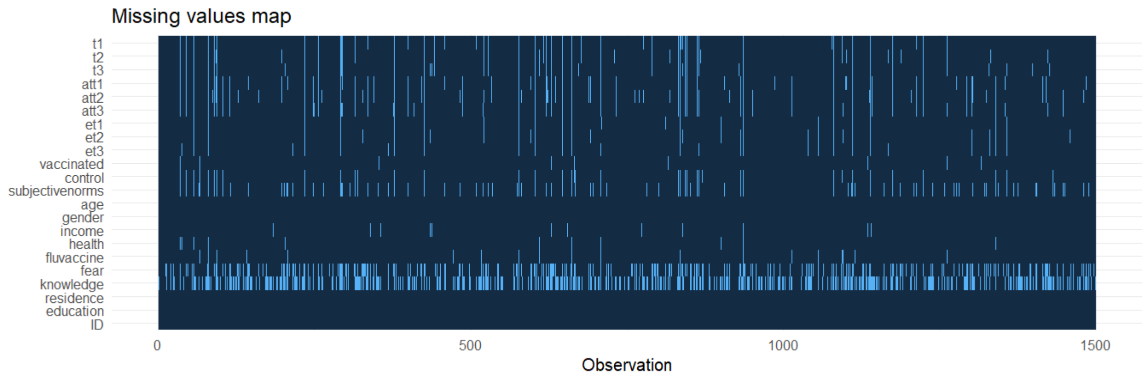

MCAR test

```
mcar_test(full)
```

```
# A tibble: 1 × 4
  statistic    df      p.value missing.patterns
      <dbl> <dbl>        <dbl>            <int>
1     3147.  2776 0.000000872              161
```

COVID-19-related knowledge

```
model <- glm(knowledge_miss ~  age + gender + income + education + health + residence, fami
ly = "binomial", data = full)
summary(model)
```

```
Call:
glm(formula = knowledge_miss ~ age + gender + income + education +
    health + residence, family = "binomial", data = full)

Coefficients:
                  Estimate Std. Error z value Pr(>|z|)
(Intercept)      -0.021170   0.387732  -0.055  0.95646
age               0.004745   0.003632   1.306  0.19142
genderMale       -0.330039   0.116267  -2.839  0.00453 **
income           -0.055859   0.059902  -0.932  0.35108
educationMiddle  -0.083322   0.146805  -0.568  0.57033
educationUpper   -0.315345   0.187526  -1.682  0.09264 .
health           -0.028905   0.065167  -0.444  0.65736
residenceTown    -0.117022   0.150383  -0.778  0.43648
residenceVillage -0.282895   0.166785  -1.696  0.08985 .
---
Signif. codes:  0 ‘***’ 0.001 ‘**’ 0.01 ‘*’ 0.05 ‘.’ 0.1 ‘ ’ 1

(Dispersion parameter for binomial family taken to be 1)

    Null deviance: 1959.5  on 1471  degrees of freedom
Residual deviance: 1942.7  on 1463  degrees of freedom
  (28 observations deleted due to missingness)
AIC: 1960.7

Number of Fisher Scoring iterations: 4
```

Perceived COVID-19 risk

```
model <- glm(risk_miss ~  age + gender + income + education + health + residence, family =
"binomial", data = full)
summary(model)
```

```
Call:
glm(formula = risk_miss ~ age + gender + income + education +
```

```
    health + residence, family = "binomial", data = full)

Coefficients:
                  Estimate Std. Error z value Pr(>|z|)  
(Intercept)      -0.571883   0.439932  -1.300   0.1936  
age               0.009930   0.004188   2.371   0.0177 *
genderMale       -0.255755   0.134240  -1.905   0.0568 .
income           -0.103870   0.068765  -1.511   0.1309  
educationMiddle  -0.245958   0.164339  -1.497   0.1345  
educationUpper   -0.306893   0.212195  -1.446   0.1481  
health           -0.080512   0.074839  -1.076   0.2820  
residenceTown    -0.325317   0.172786  -1.883   0.0597 .
residenceVillage -0.051867   0.186239  -0.278   0.7806  
---
Signif. codes:  0 '***' 0.001 '**' 0.01 '*' 0.05 '.' 0.1 ' ' 1

(Dispersion parameter for binomial family taken to be 1)

    Null deviance: 1598.3  on 1471  degrees of freedom
Residual deviance: 1574.8  on 1463  degrees of freedom
  (28 observations deleted due to missingness)
AIC: 1592.8

Number of Fisher Scoring iterations: 4
```

Subjective norms

```
model <- glm(subjectivenorms_miss ~  age + gender + income + education + health + residence
, family = "binomial", data = full)
summary(model)
```

```
Call:
glm(formula = subjectivenorms_miss ~ age + gender + income + 
    education + health + residence, family = "binomial", data = full)

Coefficients:
                  Estimate Std. Error z value Pr(>|z|)  
(Intercept)      -1.044346   0.635334  -1.644    0.100  
age              -0.008445   0.006204  -1.361    0.173  
genderMale        0.008396   0.198302   0.042    0.966  
income           -0.128170   0.100375  -1.277    0.202  
educationMiddle  -0.297744   0.232165  -1.282    0.200  
educationUpper   -0.686291   0.330755  -2.075    0.038 *
health            0.017963   0.109698   0.164    0.870  
residenceTown    -0.242455   0.251042  -0.966    0.334  
residenceVillage -0.402407   0.283167  -1.421    0.155  
---
Signif. codes:  0 '***' 0.001 '**' 0.01 '*' 0.05 '.' 0.1 ' ' 1

(Dispersion parameter for binomial family taken to be 1)

    Null deviance: 879.14  on 1471  degrees of freedom
Residual deviance: 869.01  on 1463  degrees of freedom
  (28 observations deleted due to missingness)
AIC: 887.01

Number of Fisher Scoring iterations: 5
```

Attitude ATT2

```
model <- glm(att2_miss ~  age + gender + income + education + health + residence, family =
"binomial", data = full)
summary(model)
```

```
Call:
glm(formula = att2_miss ~ age + gender + income + education + 
    health + residence, family = "binomial", data = full)
```

```
Coefficients:
                 Estimate Std. Error z value Pr(>|z|)
(Intercept)      -1.14554    0.71746  -1.597   0.1103
age              -0.01314    0.00708  -1.856   0.0634 .
genderMale       -0.23865    0.22900  -1.042   0.2973
income           -0.12985    0.11276  -1.152   0.2495
educationMiddle  -0.36404    0.25478  -1.429   0.1530
educationUpper   -0.81672    0.38356  -2.129   0.0332 *
health            0.03917    0.12362   0.317   0.7514
residenceTown    -0.15445    0.29944  -0.516   0.6060
residenceVillage -0.13771    0.32408  -0.425   0.6709
---
Signif. codes:  0 ‘***’ 0.001 ‘**’ 0.01 ‘*’ 0.05 ‘.’ 0.1 ‘ ’ 1

(Dispersion parameter for binomial family taken to be 1)

    Null deviance: 730.89  on 1471  degrees of freedom
Residual deviance: 715.57  on 1463  degrees of freedom
  (28 observations deleted due to missingness)
AIC: 733.57

Number of Fisher Scoring iterations: 5
```
