# Supplementary material for: Trust in science, knowledge and risk perception as predictors of COVID-19 vaccination: application of an extended Theory of Planned Behavior model in Hungary
Source: BMC Public Health. 2026 Feb 3;26:774. doi: 10.1186/s12889-026-26421-5 (PMC12955181; doi:10.1186/s12889-026-26421-5)
Supplement: Supplementary file 7 — Additional file 7. Structural model (cat. ‘Not yet but I am planning to’ excluded). [file 12889_2026_26421_MOESM7_ESM.pdf]

Structural model (cat. ‘Not yet but I am planning to’ excluded)

```
m <- '
    # Measurement model
          t =~ t1 + t2 + t3
          attitude =~ att1 + att2 + att3
          et =~ et1 + et2 + et3

    # Regression models
       vaccinated2 ~ attitude + control + subjectivenorms
       attitude ~ age_sc + gender + edu_low + edu_middle + income +
                   health + fluvaccine + risk +
                   t + knowledge + et
       t ~ age_sc + gender + edu_low + edu_middle + income +
           et + risk + knowledge
       '

model <- lavaan::sem(m, data=data2, estimator = "WLSMV")
summary(model, fit.measures = T, standardized = T)
```

```
lavaan 0.6.18.1972 ended normally after 167 iterations

  Estimator                                         DWLS
  Optimization method                             NLMINB
  Number of model parameters                         107

  Number of observations                             753

Model Test User Model:
                                              Standard      Scaled
  Test Statistic                               244.086     325.359
  Degrees of freedom                               124         124
  P-value (Chi-square)                           0.000       0.000
  Scaling correction factor                                  0.904
  Shift parameter                                           55.360
    simple second-order correction

Model Test Baseline Model:

  Test statistic                             10009.110    4257.995
  Degrees of freedom                               155         155
  P-value                                        0.000       0.000
  Scaling correction factor                                  2.402

User Model versus Baseline Model:

  Comparative Fit Index (CFI)                    0.988       0.951
  Tucker-Lewis Index (TLI)                       0.985       0.939

  Robust Comparative Fit Index (CFI)                         0.988
  Robust Tucker-Lewis Index (TLI)                            0.985

Root Mean Square Error of Approximation:

  RMSEA                                          0.036       0.046
  90 Percent confidence interval - lower         0.029       0.040
  90 Percent confidence interval - upper         0.042       0.053
  P-value H_0: RMSEA <= 0.050                    1.000       0.821
  P-value H_0: RMSEA >= 0.080                    0.000       0.000

  Robust RMSEA                                               0.044
  90 Percent confidence interval - lower                     0.038
  90 Percent confidence interval - upper                     0.050
  P-value H_0: Robust RMSEA <= 0.050                         0.948
  P-value H_0: Robust RMSEA >= 0.080                         0.000

Standardized Root Mean Square Residual:

  SRMR                                           0.039       0.039
```

```
Parameter Estimates:

  Standard errors                             Robust.sem
  Information                                   Expected
  Information saturated (h1) model          Unstructured

Latent Variables:
                   Estimate  Std.Err  z-value  P(>|z|)   Std.lv  Std.all
  t =~
    t1                1.000                               1.121    0.876
    t2                1.028    0.029   35.264    0.000    1.153    0.934
    t3                0.810    0.038   21.306    0.000    0.908    0.732
  attitude =~
    att1              1.000                               1.395    0.934
    att2              0.834    0.023   36.798    0.000    1.163    0.799
    att3              0.983    0.020   49.249    0.000    1.371    0.901
  et =~
    et1               1.000                               1.240    0.718
    et2               1.018    0.087   11.639    0.000    1.262    0.781
    et3               1.095    0.085   12.913    0.000    1.357    0.767

Regressions:
                   Estimate  Std.Err  z-value  P(>|z|)   Std.lv  Std.all
  vaccinated2 ~
    attitude          0.197    0.009   20.881    0.000    0.274    0.630
    control          -0.065    0.010   -6.711    0.000   -0.065   -0.194
    subjectivenrms    0.018    0.004    4.017    0.000    0.018    0.099
  attitude ~
    age_sc            0.002    0.002    0.879    0.379    0.001    0.025
    gender            0.145    0.069    2.083    0.037    0.104    0.052
    edu_low           0.131    0.123    1.068    0.286    0.094    0.034
    edu_middle        0.064    0.093    0.688    0.491    0.046    0.022
    income            0.013    0.038    0.348    0.728    0.009    0.009
    health            0.007    0.044    0.164    0.870    0.005    0.005
    fluvaccine        0.233    0.036    6.423    0.000    0.167    0.180
    risk              0.095    0.018    5.168    0.000    0.068    0.151
    t                 0.636    0.065    9.850    0.000    0.511    0.511
    knowledge         0.364    0.058    6.230    0.000    0.261    0.268
    et               -0.009    0.044   -0.203    0.839   -0.008   -0.008
  t ~
    age_sc            0.008    0.002    3.532    0.000    0.007    0.118
    gender           -0.020    0.075   -0.265    0.791   -0.018   -0.009
    edu_low          -0.236    0.131   -1.803    0.071   -0.211   -0.077
    edu_middle       -0.293    0.088   -3.335    0.001   -0.261   -0.127
    income            0.132    0.041    3.237    0.001    0.118    0.116
    et                0.279    0.047    5.948    0.000    0.308    0.308
    risk              0.039    0.019    2.076    0.038    0.035    0.078
    knowledge         0.519    0.040   12.972    0.000    0.463    0.475
```
