# Supplementary material for: Trust in science, knowledge and risk perception as predictors of COVID-19 vaccination: application of an extended Theory of Planned Behavior model in Hungary
Source: BMC Public Health. 2026 Feb 3;26:774. doi: 10.1186/s12889-026-26421-5 (PMC12955181; doi:10.1186/s12889-026-26421-5)
Supplement: Supplementary file 8 — Additional file 8. Sub-group analysis. [file 12889_2026_26421_MOESM8_ESM.pdf]

Sub-group analysis

To test if our results hold in various sample sub-groups, we performed a sub-group analysis taking into account age (young [standardized score <= -1], middle aged [standardized score between -1 and +1], older [standardized score >= +1]), gender (male, female), and the level of education (low, middle, upper). Full models are reported below; here we limit the discussion to the effects of trust in science, perceived COVID-19 risk, and COVID-19 knowledge. Starting with trust in science, one figure stands out in particular: contrary to the groups of middle (β = 0.588, SE = 0.065) and upper education (β = 0.534, SE = 0.140), in the low-education sub-sample, trust in science has no effect on COVID-19 vaccination attitudes (β = 0.007, SE = 0.310). With regard to the difference between genders, trust seems to affect COVID-19 vaccine attitudes for women (β = 0.495, SE = 0.090) to a greater extent than for men (β = 0.370, SE = 0.130). Furthermore, while the effect of trust is about the same for younger (β = 0.471, SE = 0.269) and middle-aged (β = 0.457, SE = 0.095) respondents, we find limited support for the effect of trust in science on vaccination attitudes in the older sub-sample (β = 0.284, SE = 0.145).

Moving on with the effect of COVID-19 knowledge, we see quite a notable variation in how it relates to trust in science and COVID-19 vaccine attitudes. First, knowledge has a larger effect on trust for women (β = 0.401, SE = 0.053) than for men (β = 0.171, SE = 0.065). This suggests that increasing the levels of knowledge has an overall larger effect on vaccination attitudes for women due to a larger mediated effect via trust. Second, COVID-19 knowledge has no effect on trust in science in the low-education sub-sample (β = 0.080, SE = 0.115). Nevertheless, the effect of knowledge on vaccination attitudes is still notable in this group (β = 0.636, SE = 0.119), indicating that increasing knowledge may strengthen positive attitudes toward vaccination – and thus vaccine acceptance. Third, compared to the low- (β = 0.636, SE = 0.119) and high-education (β = 0.623, SE = 0.226) groups, the effect of knowledge on vaccine attitudes is weaker among respondents with mid-level education (β = 0.231, SE = 0.048). Fourth, our figures show that knowledge is most important for older people in increasing both trust (β = 0.547, SE = 0.105) and positive attitudes towards vaccination (β = 0.922, SE = 0.150). Consequently, knowledge campaigns are most successful when targeted to this age cohort.

Last but not least, the effect of risk shows the most stability among our key variables – with a few exceptions. We note first that risk affects trust in science for men (β = 0.281, SE = 0.029) more than for women (β = 0.152, SE = 0.025). Second, the effect of risk on trust is slightly less prominent in the group of low education (β = 0.168, SE = 0.042) than in others (middle: β = 0.212, SE = 0.024; higher: β = 0.195, SE = 0.032). Third, risk has a somewhat stronger correlation with trust for older respondents (β = 0.118, SE = 0.041), but a smaller effect on COVID-19 vaccine attitudes (β = 0.147, SE = 0.033) than middle-aged individuals.

Age sub-groups

```
# Subsetting by age
young <- data[which(data$age_sc <= -1),]
middleaged <- data[which(data$age_sc > -1 & data$age_sc < 1),]
old <- data[which(data$age_sc >= 1),]

# Model for the youngest cohort
m <- '
    # Measurement model
          t =~ t1 + t2 + t3
          attitude =~ att1 + att2 + att3
          et =~ et1 + et2 + et3

    # Regression models
       vaccinated ~ attitude + control + subjectivenorms
       attitude ~ gender + edu_low + edu_middle + income +
                   health + fluvaccine + risk +
                   t + knowledge + et
       t ~ gender + edu_low + edu_middle + income +
          et + risk + knowledge
       '
model <- lavaan::sem(m, data=young, estimator = "WLSMV")
summary(model, fit.measures = T, standardized = T)
```

```
lavaan 0.6-18.2125 ended normally after 663 iterations

  Estimator                                         DWLS
  Optimization method                             NLMINB
  Number of model parameters                          94

                                                    Used       Total
  Number of observations                             142         287

Model Test User Model:
                                                Standard      Scaled
  Test Statistic                                 132.738     192.967
  Degrees of freedom                                 116         116
  P-value (Chi-square)                             0.137       0.000
  Scaling correction factor                                    0.991
  Shift parameter                                             59.002
    simple second-order correction

Model Test Baseline Model:

  Test statistic                                1632.281     735.116
  Degrees of freedom                                 145         145
  P-value                                          0.000       0.000
  Scaling correction factor                                    2.520

User Model versus Baseline Model:

  Comparative Fit Index (CFI)                      0.989       0.870
  Tucker-Lewis Index (TLI)                         0.986       0.837

  Robust Comparative Fit Index (CFI)                           0.949
  Robust Tucker-Lewis Index (TLI)                              0.936

Root Mean Square Error of Approximation:

  RMSEA                                            0.032       0.069
  90 Percent confidence interval - lower           0.000       0.051
  90 Percent confidence interval - upper           0.055       0.085
  P-value H_0: RMSEA <= 0.050                      0.892       0.041
  P-value H_0: RMSEA >= 0.080                      0.000       0.137

  Robust RMSEA                                                 0.068
  90 Percent confidence interval - lower                       0.051
```

```
  90 Percent confidence interval - upper                     0.085
  P-value H_0: Robust RMSEA <= 0.050                         0.043
  P-value H_0: Robust RMSEA >= 0.080                         0.129

Standardized Root Mean Square Residual:

  SRMR                                           0.070       0.070

Parameter Estimates:

  Standard errors                           Robust.sem
  Information                                 Expected
  Information saturated (h1) model        Unstructured

Latent Variables:
                   Estimate  Std.Err  z-value  P(>|z|)   Std.lv  Std.all
  t =~
    t1                1.000                               1.130    0.873
    t2                1.031    0.060   17.293    0.000    1.164    0.939
    t3                0.724    0.097    7.486    0.000    0.818    0.661
  attitude =~
    att1              1.000                               1.234    0.891
    att2              0.839    0.061   13.715    0.000    1.036    0.764
    att3              1.035    0.070   14.705    0.000    1.278    0.891
  et =~
    et1               1.000                               1.435    0.802
    et2               0.798    0.182    4.392    0.000    1.146    0.708
    et3               0.777    0.171    4.548    0.000    1.115    0.663

Regressions:
                   Estimate  Std.Err  z-value  P(>|z|)   Std.lv  Std.all
  vaccinated ~
    attitude          0.227    0.025    8.967    0.000    0.281    0.580
    control          -0.108    0.022   -4.973    0.000   -0.108   -0.322
    subjectivenrms    0.018    0.013    1.339    0.181    0.018    0.090
  attitude ~
    gender            0.755    0.925    0.816    0.414    0.612    0.267
    edu_low           9.329    9.936    0.939    0.348    7.558    2.581
    edu_middle        6.689    6.969    0.960    0.337    5.419    2.513
    income            0.394    0.467    0.843    0.399    0.319    0.299
    health            0.107    0.097    1.108    0.268    0.087    0.076
    fluvaccine        0.193    0.139    1.384    0.166    0.156    0.105
    risk              0.112    0.192    0.580    0.562    0.090    0.182
    t                 0.471    0.269    1.753    0.080    0.431    0.431
    knowledge         1.570    1.436    1.094    0.274    1.272    1.302
    et                0.010    0.113    0.087    0.931    0.011    0.011
  t ~
    gender           -0.660    1.037   -0.637    0.524   -0.585   -0.255
    edu_low         -10.971   10.161   -1.080    0.280   -9.710   -3.315
    edu_middle       -7.579    7.222   -1.050    0.294   -6.708   -3.110
    income           -0.304    0.514   -0.591    0.554   -0.269   -0.252
    et                0.266    0.101    2.626    0.009    0.338    0.338
    risk              0.382    0.173    2.207    0.027    0.338    0.683
    knowledge        -1.241    1.508   -0.823    0.410   -1.098   -1.125
```

```
# Model for the middle cohort
m <- '
    # Measurement model
          t =~ t1 + t2 + t3
          attitude =~ att1 + att2 + att3
          et =~ et1 + et2 + et3

    # Regression models
       vaccinated ~ attitude + control + subjectivenorms
       attitude ~ gender + edu_low + edu_middle + income +
```

```
                   health + fluvaccine + risk +
                   t + knowledge + et
       t ~ gender + edu_low + edu_middle + income +
          et + risk + knowledge
       '
model <- lavaan::sem(m, data=middleaged, estimator = "WLSMV")
summary(model, fit.measures = T, standardized = T)
```

```
lavaan 0.6-18.2125 ended normally after 117 iterations

  Estimator                                         DWLS
  Optimization method                             NLMINB
  Number of model parameters                          94

                                                    Used       Total
  Number of observations                             453         878

Model Test User Model:
                                                Standard      Scaled
  Test Statistic                                 158.613     226.715
  Degrees of freedom                                 116         116
  P-value (Chi-square)                             0.005       0.000
  Scaling correction factor                                    0.916
  Shift parameter                                             53.521
    simple second-order correction

Model Test Baseline Model:

  Test statistic                                6147.183    2567.719
  Degrees of freedom                                 145         145
  P-value                                          0.000       0.000
  Scaling correction factor                                    2.477

User Model versus Baseline Model:

  Comparative Fit Index (CFI)                      0.993       0.954
  Tucker-Lewis Index (TLI)                         0.991       0.943

  Robust Comparative Fit Index (CFI)                           0.983
  Robust Tucker-Lewis Index (TLI)                              0.979

Root Mean Square Error of Approximation:

  RMSEA                                            0.029       0.046
  90 Percent confidence interval - lower           0.016       0.037
  90 Percent confidence interval - upper           0.039       0.055
  P-value H_0: RMSEA <= 0.050                      1.000       0.766
  P-value H_0: RMSEA >= 0.080                      0.000       0.000

  Robust RMSEA                                                 0.044
  90 Percent confidence interval - lower                       0.035
  90 Percent confidence interval - upper                       0.052
  P-value H_0: Robust RMSEA <= 0.050                           0.876
  P-value H_0: Robust RMSEA >= 0.080                           0.000

Standardized Root Mean Square Residual:

  SRMR                                             0.043       0.043

Parameter Estimates:

  Standard errors                             Robust.sem
  Information                                   Expected
  Information saturated (h1) model          Unstructured

Latent Variables:
                   Estimate  Std.Err  z-value  P(>|z|)   Std.lv  Std.all
```

```
  t =~
    t1                1.000                                  1.113    0.858
    t2                1.054    0.039   27.270    0.000    1.173    0.941
    t3                0.840    0.050   16.673    0.000    0.935    0.757
  attitude =~
    att1              1.000                                  1.381    0.923
    att2              0.809    0.031   26.100    0.000    1.117    0.789
    att3              0.993    0.028   36.011    0.000    1.372    0.892
  et =~
    et1               1.000                                  1.243    0.723
    et2               0.992    0.119    8.350    0.000    1.234    0.767
    et3               1.077    0.112    9.649    0.000    1.338    0.760

Regressions:
                   Estimate  Std.Err  z-value  P(>|z|)   Std.lv  Std.all
  vaccinated ~
    attitude          0.194    0.015   13.229    0.000    0.268    0.598
    control          -0.080    0.013   -6.276    0.000   -0.080   -0.237
    subjectivenrms    0.011    0.006    1.773    0.076    0.011    0.071
  attitude ~
    gender            0.242    0.083    2.923    0.003    0.176    0.088
    edu_low          -0.069    0.207   -0.335    0.738   -0.050   -0.016
    edu_middle       -0.202    0.148   -1.367    0.172   -0.146   -0.069
    income            0.019    0.049    0.382    0.702    0.014    0.014
    health            0.056    0.049    1.132    0.258    0.040    0.037
    fluvaccine        0.150    0.046    3.252    0.001    0.109    0.105
    risk              0.225    0.037    6.059    0.000    0.163    0.346
    t                 0.457    0.095    4.812    0.000    0.369    0.369
    knowledge         0.347    0.054    6.437    0.000    0.251    0.255
    et                0.081    0.065    1.250    0.211    0.073    0.073
  t ~
    gender            0.002    0.090    0.023    0.982    0.002    0.001
    edu_low          -0.629    0.206   -3.049    0.002   -0.565   -0.186
    edu_middle       -0.482    0.127   -3.807    0.000   -0.434   -0.203
    income            0.148    0.050    2.960    0.003    0.133    0.135
    et                0.315    0.060    5.255    0.000    0.352    0.352
    risk              0.234    0.025    9.429    0.000    0.210    0.446
    knowledge         0.296    0.053    5.607    0.000    0.266    0.271
```

```
# Model for the oldest cohort
m <- '
    # Measurement model
          t =~ t1 + t2 + t3
          attitude =~ att1 + att2 + att3
          et =~ et1 + et2 + et3

    # Regression models
       vaccinated ~ attitude + control + subjectivenorms
       attitude ~ gender + edu_low + edu_middle + income +
                   health + fluvaccine + risk +
                   t + knowledge + et
       t ~ gender + edu_low + edu_middle + income +
          et + risk + knowledge
       '
model <- lavaan::sem(m, data=old, estimator = "WLSMV")
summary(model, fit.measures = T, standardized = T)
```

```
lavaan 0.6-18.2125 ended normally after 105 iterations

  Estimator                                         DWLS
  Optimization method                             NLMINB
  Number of model parameters                          94

                                                    Used       Total
  Number of observations                             166         335
```

```
Model Test User Model:
                                              Standard      Scaled
  Test Statistic                                87.842     149.903
  Degrees of freedom                               116         116
  P-value (Chi-square)                           0.976       0.019
  Scaling correction factor                                  0.997
  Shift parameter                                           61.818
    simple second-order correction

Model Test Baseline Model:

  Test statistic                              1613.198     603.791
  Degrees of freedom                               145         145
  P-value                                        0.000       0.000
  Scaling correction factor                                  3.200

User Model versus Baseline Model:

  Comparative Fit Index (CFI)                    1.000       0.926
  Tucker-Lewis Index (TLI)                       1.024       0.908

  Robust Comparative Fit Index (CFI)                         0.977
  Robust Tucker-Lewis Index (TLI)                            0.971

Root Mean Square Error of Approximation:

  RMSEA                                          0.000       0.042
  90 Percent confidence interval - lower         0.000       0.018
  90 Percent confidence interval - upper         0.000       0.060
  P-value H_0: RMSEA <= 0.050                    1.000       0.744
  P-value H_0: RMSEA >= 0.080                    0.000       0.000

  Robust RMSEA                                               0.042
  90 Percent confidence interval - lower                     0.018
  90 Percent confidence interval - upper                     0.060
  P-value H_0: Robust RMSEA <= 0.050                         0.746
  P-value H_0: Robust RMSEA >= 0.080                         0.000

Standardized Root Mean Square Residual:

  SRMR                                           0.054       0.054

Parameter Estimates:

  Standard errors                             Robust.sem
  Information                                   Expected
  Information saturated (h1) model          Unstructured

Latent Variables:
                   Estimate  Std.Err  z-value  P(>|z|)   Std.lv  Std.all
  t =~
    t1                1.000                               0.966    0.896
    t2                0.949    0.082   11.627    0.000    0.917    0.896
    t3                0.698    0.086    8.120    0.000    0.674    0.624
  attitude =~
    att1              1.000                               1.183    0.927
    att2              1.021    0.056   18.167    0.000    1.209    0.836
    att3              0.959    0.039   24.623    0.000    1.135    0.906
  et =~
    et1               1.000                               1.176    0.717
    et2               1.102    0.150    7.329    0.000    1.296    0.804
    et3               1.303    0.228    5.725    0.000    1.533    0.841

Regressions:
                   Estimate  Std.Err  z-value  P(>|z|)   Std.lv  Std.all
  vaccinated ~
```

```
    attitude          0.147    0.027    5.365    0.000    0.174    0.626
    control          -0.009    0.028   -0.319    0.749   -0.009   -0.027
    subjectivenrms    0.007    0.007    1.023    0.306    0.007    0.072
  attitude ~
    gender            0.032    0.142    0.224    0.822    0.027    0.013
    edu_low          -0.046    0.145   -0.315    0.753   -0.039   -0.016
    edu_middle       -0.094    0.144   -0.655    0.512   -0.080   -0.040
    income            0.007    0.069    0.108    0.914    0.006    0.006
    health           -0.121    0.075   -1.603    0.109   -0.102   -0.087
    fluvaccine        0.043    0.051    0.853    0.394    0.037    0.047
    risk              0.147    0.033    4.413    0.000    0.125    0.251
    t                 0.284    0.145    1.961    0.050    0.232    0.232
    knowledge         0.922    0.150    6.135    0.000    0.779    0.598
    et                0.070    0.103    0.680    0.497    0.070    0.070
  t ~
    gender           -0.159    0.134   -1.188    0.235   -0.165   -0.079
    edu_low          -0.049    0.206   -0.237    0.812   -0.051   -0.021
    edu_middle       -0.340    0.137   -2.477    0.013   -0.352   -0.175
    income            0.119    0.076    1.571    0.116    0.123    0.110
    et                0.239    0.081    2.935    0.003    0.291    0.291
    risk              0.118    0.041    2.898    0.004    0.122    0.246
    knowledge         0.574    0.105    5.457    0.000    0.594    0.456
```

Education sub-groups

```
# Subsetting by education
lowedu <- data[which(data$education == "Lower"),]
middleedu <- data[which(data$education == "Middle"),]
highedu <- data[which(data$education == "Upper"),]

# Model for the lowest education group
m <- '
    # Measurement model
          t =~ t1 + t2 + t3
          attitude =~ att1 + att2 + att3
          et =~ et1 + et2 + et3

    # Regression models
       vaccinated ~ attitude + control + subjectivenorms
       attitude ~ age_sc + gender + income +
                   health + fluvaccine + risk +
                   t + knowledge + et
       t ~ age_sc + gender + income +
          et + risk + knowledge
       '
model <- lavaan::sem(m, data=lowedu, estimator = "WLSMV")
summary(model, fit.measures = T, standardized = T)
```

```
lavaan 0.6-18.2125 ended normally after 87 iterations

  Estimator                                         DWLS
  Optimization method                             NLMINB
  Number of model parameters                          82

                                                    Used       Total
  Number of observations                             121         255

Model Test User Model:
                                                Standard      Scaled
  Test Statistic                                 102.290     158.515
  Degrees of freedom                                 108         108
  P-value (Chi-square)                             0.637       0.001
  Scaling correction factor                                    1.040
  Shift parameter                                             60.135
    simple second-order correction

Model Test Baseline Model:

  Test statistic                                2039.980     787.516
  Degrees of freedom                                 135         135
  P-value                                          0.000       0.000
  Scaling correction factor                                    2.919

User Model versus Baseline Model:

  Comparative Fit Index (CFI)                      1.000       0.923
  Tucker-Lewis Index (TLI)                         1.004       0.903

  Robust Comparative Fit Index (CFI)                           0.972
  Robust Tucker-Lewis Index (TLI)                              0.966

Root Mean Square Error of Approximation:

  RMSEA                                            0.000       0.062
  90 Percent confidence interval - lower           0.000       0.040
  90 Percent confidence interval - upper           0.041       0.082
  P-value H_0: RMSEA <= 0.050                      0.987       0.164
  P-value H_0: RMSEA >= 0.080                      0.000       0.077

  Robust RMSEA                                                 0.064
  90 Percent confidence interval - lower                       0.041
```

```
  90 Percent confidence interval - upper                     0.084
  P-value H_0: Robust RMSEA <= 0.050                         0.148
  P-value H_0: Robust RMSEA >= 0.080                         0.098

Standardized Root Mean Square Residual:

  SRMR                                           0.070       0.070

Parameter Estimates:

  Standard errors                             Robust.sem
  Information                                   Expected
  Information saturated (h1) model           Unstructured

Latent Variables:
                   Estimate  Std.Err  z-value  P(>|z|)   Std.lv  Std.all
  t =~
    t1                1.000                               1.152    0.829
    t2                1.079    0.076   14.275    0.000    1.244    0.929
    t3                0.875    0.092    9.482    0.000    1.008    0.746
  attitude =~
    att1              1.000                               1.509    0.945
    att2              0.814    0.048   17.088    0.000    1.227    0.799
    att3              0.868    0.054   16.022    0.000    1.309    0.850
  et =~
    et1               1.000                               1.111    0.637
    et2               1.578    0.317    4.974    0.000    1.752    1.025
    et3               1.050    0.196    5.367    0.000    1.166    0.663

Regressions:
                   Estimate  Std.Err  z-value  P(>|z|)   Std.lv  Std.all
  vaccinated ~
    attitude          0.215    0.027    7.848    0.000    0.325    0.683
    control          -0.073    0.030   -2.424    0.015   -0.073   -0.181
    subjectivenrms   -0.001    0.015   -0.076    0.939   -0.001   -0.007
  attitude ~
    age_sc           -0.030    0.131   -0.230    0.818   -0.020   -0.022
    gender            0.346    0.174    1.991    0.047    0.229    0.115
    income            0.099    0.172    0.577    0.564    0.066    0.069
    health            0.042    0.096    0.439    0.661    0.028    0.025
    fluvaccine        0.180    0.103    1.752    0.080    0.120    0.130
    risk              0.270    0.069    3.924    0.000    0.179    0.451
    t                 0.007    0.310    0.023    0.982    0.005    0.005
    knowledge         0.636    0.119    5.338    0.000    0.421    0.450
    et                0.377    0.242    1.560    0.119    0.277    0.277
  t ~
    age_sc            0.249    0.130    1.914    0.056    0.216    0.236
    gender           -0.005    0.191   -0.025    0.980   -0.004   -0.002
    income            0.408    0.084    4.877    0.000    0.354    0.371
    et                0.434    0.119    3.644    0.000    0.418    0.418
    risk              0.168    0.042    4.003    0.000    0.146    0.367
    knowledge         0.080    0.115    0.698    0.485    0.069    0.074
```

```
# Total effect of knowledge for the lowest education group
m <- '
   # Measurement model
         t =~ t1 + t2 + t3
         attitude =~ att1 + att2 + att3
         et =~ et1 + et2 + et3

   # Regression models
      vaccinated ~ d*attitude + control + subjectivenorms
      attitude ~ age_sc + gender + income +
                 health + fluvaccine + risk +
                 c*t + b*knowledge + et
      t ~ age_sc + gender + income +
```

```
        et + risk + a*knowledge

   # Total effect of knowledge on vaccination
      eff:= a*c*d + b*d
      '
model <- lavaan::sem(m, data=lowedu, estimator = "DWLS", se = "bootstrap",
                     bootstrap = 1000,
                     test="scaled.shifted", verbose = T)
summary(model, fit.measures = T, standardized = T)
```

```
Defined Parameters:
                   Estimate  Std.Err  z-value  P(>|z|)   Std.lv  Std.all
    eff               0.137    0.041    3.366    0.001    0.137    0.308
```

```
# Model for the middle education group
m <- '
    # Measurement model
          t =~ t1 + t2 + t3
          attitude =~ att1 + att2 + att3
          et =~ et1 + et2 + et3

    # Regression models
       vaccinated ~ attitude + control + subjectivenorms
       attitude ~ age_sc + gender + income +
                   health + fluvaccine + risk +
                   t + knowledge + et
       t ~ age_sc + gender + income +
          et + risk + knowledge
       '
model <- lavaan::sem(m, data=middleedu, estimator = "WLSMV")
summary(model, fit.measures = T, standardized = T)
```

```
lavaan 0.6-18.2125 ended normally after 91 iterations

  Estimator                                       DWLS
  Optimization method                           NLMINB
  Number of model parameters                        82

                                                  Used       Total
  Number of observations                           474         945

Model Test User Model:
                                              Standard      Scaled
  Test Statistic                               135.007     197.301
  Degrees of freedom                               108         108
  P-value (Chi-square)                           0.040       0.000
  Scaling correction factor                                  0.925
  Shift parameter                                           51.288
    simple second-order correction

Model Test Baseline Model:

  Test statistic                              6935.998    2859.852
  Degrees of freedom                               135         135
  P-value                                        0.000       0.000
  Scaling correction factor                                  2.496

User Model versus Baseline Model:

  Comparative Fit Index (CFI)                    0.996       0.967
  Tucker-Lewis Index (TLI)                       0.995       0.959

  Robust Comparative Fit Index (CFI)                         0.996
  Robust Tucker-Lewis Index (TLI)                            0.995

Root Mean Square Error of Approximation:
```

```
  RMSEA                                         0.023       0.042
  90 Percent confidence interval - lower        0.005       0.032
  90 Percent confidence interval - upper        0.034       0.051
  P-value H_0: RMSEA <= 0.050                   1.000       0.929
  P-value H_0: RMSEA >= 0.080                   0.000       0.000

  Robust RMSEA                                              0.040
  90 Percent confidence interval - lower                    0.031
  90 Percent confidence interval - upper                    0.049
  P-value H_0: Robust RMSEA <= 0.050                        0.967
  P-value H_0: Robust RMSEA >= 0.080                        0.000

Standardized Root Mean Square Residual:

  SRMR                                          0.040       0.040

Parameter Estimates:

  Standard errors                             Robust.sem
  Information                                   Expected
  Information saturated (h1) model          Unstructured

Latent Variables:
                   Estimate  Std.Err  z-value  P(>|z|)   Std.lv  Std.all
  t =~
    t1                1.000                               1.120    0.878
    t2                1.024    0.036   28.794    0.000    1.148    0.931
    t3                0.858    0.046   18.797    0.000    0.962    0.766
  attitude =~
    att1              1.000                               1.343    0.919
    att2              0.833    0.033   25.616    0.000    1.118    0.786
    att3              1.030    0.025   41.561    0.000    1.384    0.914
  et =~
    et1               1.000                               1.244    0.715
    et2               0.987    0.111    8.912    0.000    1.228    0.751
    et3               1.139    0.112   10.133    0.000    1.416    0.782

Regressions:
                   Estimate  Std.Err  z-value  P(>|z|)   Std.lv  Std.all
  vaccinated ~
    attitude          0.188    0.014   13.319    0.000    0.253    0.567
    control          -0.090    0.012   -7.617    0.000   -0.090   -0.278
    subjectivenrms    0.025    0.006    4.080    0.000    0.025    0.158
  attitude ~
    age_sc           -0.039    0.043   -0.916    0.360   -0.029   -0.027
    gender            0.130    0.081    1.604    0.109    0.097    0.048
    income            0.013    0.045    0.297    0.767    0.010    0.010
    health            0.047    0.041    1.139    0.255    0.035    0.032
    fluvaccine        0.135    0.040    3.411    0.001    0.100    0.104
    risk              0.207    0.029    7.131    0.000    0.154    0.333
    t                 0.588    0.065    9.002    0.000    0.490    0.490
    knowledge         0.231    0.048    4.848    0.000    0.172    0.176
    et               -0.028    0.050   -0.554    0.579   -0.025   -0.025
  t ~
    age_sc            0.021    0.052    0.407    0.684    0.019    0.018
    gender           -0.025    0.092   -0.277    0.782   -0.023   -0.011
    income            0.145    0.049    2.971    0.003    0.130    0.125
    et                0.267    0.060    4.449    0.000    0.296    0.296
    risk              0.212    0.024    8.924    0.000    0.189    0.408
    knowledge         0.349    0.048    7.221    0.000    0.312    0.319
```

```
# Total effect of knowledge for the middle education group
m <- '
   # Measurement model
         t =~ t1 + t2 + t3
```

```
        attitude =~ att1 + att2 + att3
        et =~ et1 + et2 + et3

  # Regression models
     vaccinated ~ d*attitude + control + subjectivenorms
     attitude ~ age_sc + gender + income +
                 health + fluvaccine + risk +
                 c*t + b*knowledge + et
     t ~ age_sc + gender + income +
        et + risk + a*knowledge

  # Total effect of knowledge on vaccination
     eff:= a*c*d + b*d
     '
model <- lavaan::sem(m, data=middleedu, estimator = "DWLS", se = "bootstrap",
                     bootstrap = 1000,
                     test="scaled.shifted", verbose = T)
summary(model, fit.measures = T, standardized = T)
```

```
Defined Parameters:
                   Estimate  Std.Err  z-value  P(>|z|)   Std.lv  Std.all
    eff               0.082    0.014    5.936    0.000    0.082    0.188
```

```
# Model for the highest education group
m <- '
    # Measurement model
        t =~ t1 + t2 + t3
        attitude =~ att1 + att2 + att3
        et =~ et1 + et2 + et3

    # Regression models
       vaccinated ~ attitude + control + subjectivenorms
       attitude ~ age_sc + gender + income +
                   health + fluvaccine + risk +
                   t + knowledge + et
       t ~ age_sc + gender + income +
          et + risk + knowledge
       '
model <- lavaan::sem(m, data=highedu, estimator = "WLSMV")
summary(model, fit.measures = T, standardized = T)
```

```
lavaan 0.6-18.2125 ended normally after 80 iterations

  Estimator                                       DWLS
  Optimization method                           NLMINB
  Number of model parameters                        82

                                                  Used       Total
  Number of observations                           166         300

Model Test User Model:
                                              Standard      Scaled
  Test Statistic                                78.682     135.095
  Degrees of freedom                               108         108
  P-value (Chi-square)                           0.985       0.040
  Scaling correction factor                                  0.988
  Shift parameter                                           55.425
    simple second-order correction

Model Test Baseline Model:

  Test statistic                              1785.514     722.834
  Degrees of freedom                               135         135
  P-value                                        0.000       0.000
  Scaling correction factor                                  2.808

User Model versus Baseline Model:
```

```
  Comparative Fit Index (CFI)                    1.000       0.954
  Tucker-Lewis Index (TLI)                       1.022       0.942

  Robust Comparative Fit Index (CFI)                         0.984
  Robust Tucker-Lewis Index (TLI)                            0.980

Root Mean Square Error of Approximation:

  RMSEA                                          0.000       0.039
  90 Percent confidence interval - lower         0.000       0.009
  90 Percent confidence interval - upper         0.000       0.058
  P-value H_0: RMSEA <= 0.050                    1.000       0.808
  P-value H_0: RMSEA >= 0.080                    0.000       0.000

  Robust RMSEA                                               0.039
  90 Percent confidence interval - lower                     0.009
  90 Percent confidence interval - upper                     0.058
  P-value H_0: Robust RMSEA <= 0.050                         0.816
  P-value H_0: Robust RMSEA >= 0.080                         0.000

Standardized Root Mean Square Residual:

  SRMR                                           0.051       0.051

Parameter Estimates:

  Standard errors                           Robust.sem
  Information                                 Expected
  Information saturated (h1) model        Unstructured

Latent Variables:
                   Estimate  Std.Err  z-value  P(>|z|)   Std.lv  Std.all
  t =~
    t1                1.000                               0.769    0.778
    t2                1.282    0.108   11.893    0.000    0.985    0.945
    t3                0.958    0.160    6.006    0.000    0.736    0.690
  attitude =~
    att1              1.000                               1.251    0.913
    att2              0.961    0.050   19.167    0.000    1.203    0.856
    att3              0.988    0.051   19.523    0.000    1.237    0.898
  et =~
    et1               1.000                               1.160    0.720
    et2               0.774    0.117    6.619    0.000    0.897    0.632
    et3               1.229    0.184    6.665    0.000    1.426    0.885

Regressions:
                   Estimate  Std.Err  z-value  P(>|z|)   Std.lv  Std.all
  vaccinated ~
    attitude          0.174    0.024    7.159    0.000    0.218    0.654
    control           0.000    0.021    0.006    0.996    0.000    0.000
    subjectivenrms    0.006    0.006    0.991    0.322    0.006    0.054
  attitude ~
    age_sc           -0.131    0.092   -1.433    0.152   -0.105   -0.107
    gender            0.154    0.142    1.085    0.278    0.123    0.061
    income            0.074    0.065    1.143    0.253    0.059    0.050
    health           -0.083    0.115   -0.719    0.472   -0.066   -0.048
    fluvaccine        0.192    0.060    3.191    0.001    0.153    0.177
    risk              0.201    0.051    3.938    0.000    0.161    0.275
    t                 0.534    0.140    3.813    0.000    0.328    0.328
    knowledge         0.623    0.116    5.377    0.000    0.498    0.450
    et               -0.025    0.076   -0.327    0.744   -0.023   -0.023
  t ~
    age_sc           -0.004    0.065   -0.067    0.946   -0.006   -0.006
    gender           -0.011    0.105   -0.105    0.916   -0.014   -0.007
    income            0.004    0.060    0.067    0.947    0.005    0.004
    et                0.070    0.064    1.088    0.277    0.105    0.105
```

```
    risk              0.195    0.032    6.022    0.000    0.254    0.433
    knowledge         0.368    0.081    4.550    0.000    0.479    0.433
```

```
# Total effect of knowledge for the highest education group
m <- '
   # Measurement model
         t =~ t1 + t2 + t3
         attitude =~ att1 + att2 + att3
         et =~ et1 + et2 + et3

   # Regression models
      vaccinated ~ d*attitude + control + subjectivenorms
      attitude ~ age_sc + gender + income +
                  health + fluvaccine + risk +
                  c*t + b*knowledge + et
      t ~ age_sc + gender + income +
         et + risk + a*knowledge

   # Total effect of knowledge on vaccination
      eff:= a*c*d + b*d
      '
model <- lavaan::sem(m, data=highedu, estimator = "DWLS", se = "bootstrap",
                     bootstrap = 1000,
                     test="scaled.shifted", verbose = T)
summary(model, fit.measures = T, standardized = T)
```

```
Defined Parameters:
                   Estimate  Std.Err  z-value  P(>|z|)   Std.lv  Std.all
    eff               0.143    0.054    2.627    0.009    0.143    0.387
```

Gender sub-groups

```
# Subsetting by gender
male <- data[which(data$gender == "Male"),]
female <- data[which(data$gender == "Female"),]

# Model for male respondents
m <- '
    # Measurement model
          t =~ t1 + t2 + t3
          attitude =~ att1 + att2 + att3
          et =~ et1 + et2 + et3

    # Regression models
       vaccinated ~ attitude + control + subjectivenorms
       attitude ~ age_sc  + edu_low + edu_middle + income +
                   health + fluvaccine + risk +
                   t + knowledge + et
       t ~ age_sc + edu_low + edu_middle + income +
          et + risk + knowledge
       '
model <- lavaan::sem(m, data=male, estimator = "WLSMV")
summary(model, fit.measures = T, standardized = T)
```

```
lavaan 0.6-18.2125 ended normally after 110 iterations

  Estimator                                         DWLS
  Optimization method                             NLMINB
  Number of model parameters                          94

                                                    Used       Total
  Number of observations                             372         703

Model Test User Model:
                                                Standard      Scaled
  Test Statistic                                 138.478     210.931
  Degrees of freedom                                 116         116
  P-value (Chi-square)                             0.076       0.000
  Scaling correction factor                                    0.923
  Shift parameter                                             60.945
    simple second-order correction

Model Test Baseline Model:

  Test statistic                                5791.639    2092.341
  Degrees of freedom                                 145         145
  P-value                                          0.000       0.000
  Scaling correction factor                                    2.900

User Model versus Baseline Model:

  Comparative Fit Index (CFI)                      0.996       0.951
  Tucker-Lewis Index (TLI)                         0.995       0.939

  Robust Comparative Fit Index (CFI)                           0.996
  Robust Tucker-Lewis Index (TLI)                              0.995

Root Mean Square Error of Approximation:

  RMSEA                                            0.023       0.047
  90 Percent confidence interval - lower           0.000       0.037
  90 Percent confidence interval - upper           0.036       0.057
  P-value H_0: RMSEA <= 0.050                      1.000       0.679
  P-value H_0: RMSEA >= 0.080                      0.000       0.000

  Robust RMSEA                                                 0.045
  90 Percent confidence interval - lower                       0.035
```

```
  90 Percent confidence interval - upper                    0.055
  P-value H_0: Robust RMSEA <= 0.050                        0.790
  P-value H_0: Robust RMSEA >= 0.080                        0.000

Standardized Root Mean Square Residual:

  SRMR                                         0.045       0.045

Parameter Estimates:

  Standard errors                         Robust.sem
  Information                               Expected
  Information saturated (h1) model        Unstructured

Latent Variables:
                   Estimate  Std.Err  z-value  P(>|z|)   Std.lv  Std.all
  t =~
    t1                1.000                               1.141    0.889
    t2                1.008    0.042   23.953    0.000    1.150    0.932
    t3                0.880    0.049   18.134    0.000    1.004    0.817
  attitude =~
    att1              1.000                               1.375    0.939
    att2              0.891    0.029   30.475    0.000    1.225    0.833
    att3              1.006    0.025   40.969    0.000    1.383    0.920
  et =~
    et1               1.000                               1.200    0.699
    et2               1.020    0.127    8.022    0.000    1.224    0.768
    et3               1.223    0.145    8.449    0.000    1.468    0.826

Regressions:
                   Estimate  Std.Err  z-value  P(>|z|)   Std.lv  Std.all
  vaccinated ~
    attitude          0.159    0.017    9.450    0.000    0.219    0.567
    control          -0.067    0.014   -4.647    0.000   -0.067   -0.215
    subjectivenrms    0.019    0.007    2.833    0.005    0.019    0.138
  attitude ~
    age_sc           -0.017    0.052   -0.326    0.745   -0.012   -0.012
    edu_low          -0.223    0.174   -1.281    0.200   -0.162   -0.058
    edu_middle       -0.304    0.154   -1.967    0.049   -0.221   -0.109
    income            0.003    0.054    0.051    0.959    0.002    0.002
    health            0.005    0.052    0.104    0.917    0.004    0.004
    fluvaccine        0.161    0.037    4.392    0.000    0.117    0.134
    risk              0.232    0.050    4.668    0.000    0.169    0.359
    t                 0.370    0.130    2.853    0.004    0.307    0.307
    knowledge         0.365    0.063    5.812    0.000    0.265    0.284
    et                0.175    0.088    1.991    0.046    0.152    0.152
  t ~
    age_sc            0.082    0.061    1.336    0.181    0.072    0.068
    edu_low          -0.485    0.193   -2.508    0.012   -0.425   -0.151
    edu_middle       -0.530    0.128   -4.134    0.000   -0.465   -0.229
    income            0.184    0.052    3.542    0.000    0.161    0.158
    et                0.350    0.067    5.214    0.000    0.368    0.368
    risk              0.281    0.029    9.784    0.000    0.246    0.523
    knowledge         0.171    0.065    2.637    0.008    0.150    0.161
```

```
# Model for female respondents
m <- '
    # Measurement model
          t =~ t1 + t2 + t3
          attitude =~ att1 + att2 + att3
          et =~ et1 + et2 + et3

    # Regression models
       vaccinated ~ attitude + control + subjectivenorms
```

```
        attitude ~ age_sc  + edu_low + edu_middle + income +
                   health + fluvaccine + risk +
                   t + knowledge + et
        t ~ age_sc + edu_low + edu_middle + income +
           et + risk + knowledge
        '
model <- lavaan::sem(m, data=female, estimator = "WLSMV")
summary(model, fit.measures = T, standardized = T)
```

```
lavaan 0.6-18.2125 ended normally after 104 iterations

  Estimator                                         DWLS
  Optimization method                             NLMINB
  Number of model parameters                          94

                                                    Used       Total
  Number of observations                             389         797

Model Test User Model:
                                                Standard      Scaled
  Test Statistic                                 158.581     216.731
  Degrees of freedom                                 116         116
  P-value (Chi-square)                             0.005       0.000
  Scaling correction factor                                    0.978
  Shift parameter                                             54.502
    simple second-order correction

Model Test Baseline Model:

  Test statistic                                5171.307    2261.540
  Degrees of freedom                                 145         145
  P-value                                          0.000       0.000
  Scaling correction factor                                    2.375

User Model versus Baseline Model:

  Comparative Fit Index (CFI)                      0.992       0.952
  Tucker-Lewis Index (TLI)                         0.989       0.941

  Robust Comparative Fit Index (CFI)                           0.992
  Robust Tucker-Lewis Index (TLI)                              0.989

Root Mean Square Error of Approximation:

  RMSEA                                            0.031       0.047
  90 Percent confidence interval - lower           0.017       0.037
  90 Percent confidence interval - upper           0.042       0.057
  P-value H_0: RMSEA <= 0.050                      0.998       0.664
  P-value H_0: RMSEA >= 0.080                      0.000       0.000

  Robust RMSEA                                                 0.047
  90 Percent confidence interval - lower                       0.037
  90 Percent confidence interval - upper                       0.056
  P-value H_0: Robust RMSEA <= 0.050                           0.699
  P-value H_0: Robust RMSEA >= 0.080                           0.000

Standardized Root Mean Square Residual:

  SRMR                                             0.046       0.046

Parameter Estimates:

  Standard errors                             Robust.sem
  Information                                   Expected
  Information saturated (h1) model            Unstructured

Latent Variables:
```

```
                   Estimate  Std.Err  z-value  P(>|z|)   Std.lv  Std.all
  t =~
    t1                1.000                               1.052    0.836
    t2                1.090    0.044   24.831    0.000    1.147    0.937
    t3                0.806    0.061   13.130    0.000    0.848    0.678
  attitude =~
    att1              1.000                               1.355    0.912
    att2              0.798    0.036   21.902    0.000    1.081    0.765
    att3              0.996    0.033   30.497    0.000    1.349    0.890
  et =~
    et1               1.000                               1.233    0.717
    et2               1.037    0.124    8.393    0.000    1.279    0.788
    et3               1.006    0.105    9.604    0.000    1.241    0.720

Regressions:
                   Estimate  Std.Err  z-value  P(>|z|)   Std.lv  Std.all
  vaccinated ~
    attitude          0.221    0.015   14.959    0.000    0.299    0.642
    control          -0.074    0.014   -5.169    0.000   -0.074   -0.212
    subjectivenrms    0.010    0.007    1.539    0.124    0.010    0.061
  attitude ~
    age_sc           -0.109    0.055   -1.972    0.049   -0.081   -0.077
    edu_low          -0.129    0.186   -0.693    0.488   -0.095   -0.035
    edu_middle       -0.036    0.143   -0.253    0.800   -0.027   -0.013
    income            0.009    0.055    0.173    0.863    0.007    0.007
    health            0.055    0.056    0.989    0.323    0.041    0.037
    fluvaccine        0.177    0.055    3.237    0.001    0.130    0.127
    risk              0.228    0.035    6.601    0.000    0.168    0.355
    t                 0.495    0.090    5.507    0.000    0.385    0.385
    knowledge         0.383    0.064    5.955    0.000    0.283    0.286
    et               -0.022    0.062   -0.350    0.726   -0.020   -0.020
  t ~
    age_sc            0.046    0.055    0.836    0.403    0.044    0.042
    edu_low          -0.321    0.178   -1.804    0.071   -0.305   -0.113
    edu_middle       -0.293    0.127   -2.315    0.021   -0.279   -0.132
    income            0.134    0.052    2.564    0.010    0.128    0.124
    et                0.229    0.065    3.540    0.000    0.269    0.269
    risk              0.152    0.025    6.172    0.000    0.144    0.304
    knowledge         0.401    0.053    7.540    0.000    0.381    0.386
```
